# Supplementary material for: Benchmarking porcine pancreatic ductal organoids for drug screening applications
Source: EMBO Mol Med. 2025 Nov 4;17(12):3657–88. doi: 10.1038/s44321-025-00330-3 (PMC12686538; doi:10.1038/s44321-025-00330-3)
Supplement: Supplementary file 2 — Appendix [file 44321_2025_330_MOESM2_ESM.pdf]

Appendix

Benchmarking porcine pancreatic ductal organoids for drug screening applications

Christos Karampelias[1,2], Kaiyuan Yang[1,2], Falk J. Farkas[1,2,3], Michael Sterr[1,2], Mireia Molina van Den Bosch[1,2,3], Simone Renner[2,4,5], Janina Fuß[6], Christine von Toerne[7], Sören Franzenburg[6], Tatsuya Kin[8], Eckhard Wolf[2,4,5], Elisabeth Kemter[2,4,5], Heiko Lickert[1,2,3*]

[1]*Institute of Diabetes and Regeneration Research, Helmholtz Munich, Neuherberg, Germany*

[2]*German Center for Diabetes Research (DZD), Neuherberg, Germany*

[3]*School of Medicine and Health, Technische Universität München, Munich, Germany*

[4]*Gene Center and Center for Innovative Medical Models (CiMM), LMU Munich, Munich, Germany*

[5]*Interfaculty Center for Endocrine and Cardiovascular Disease Network Modelling and Clinical Transfer (ICONLMU), LMU Munich, Munich, Germany*

[6]*Institute of Clinical Molecular Biology, Christian-Albrechts-Universität Kiel, Kiel, Germany*

[7]*Metabolomics and Proteomics Core, Helmholtz Center Munich, German Research Center for Environmental Health, D-80939 Munich, Germany*

[8]*Clinical Islet Laboratory, University of Alberta Hospital, Edmonton, AB, Canada*

Correspondence: *heiko.lickert@helmholtz-munich.de

Table of contents

Appendix	Description	Page
Appendix Figure S1	Cell type/states classification of PPDO/HPDO.	2
Appendix Figure S2	Ligand-receptor interactions in PPDO/HPDO.	3
Appendix Figure S3	Characterization of primary porcine dataset	4

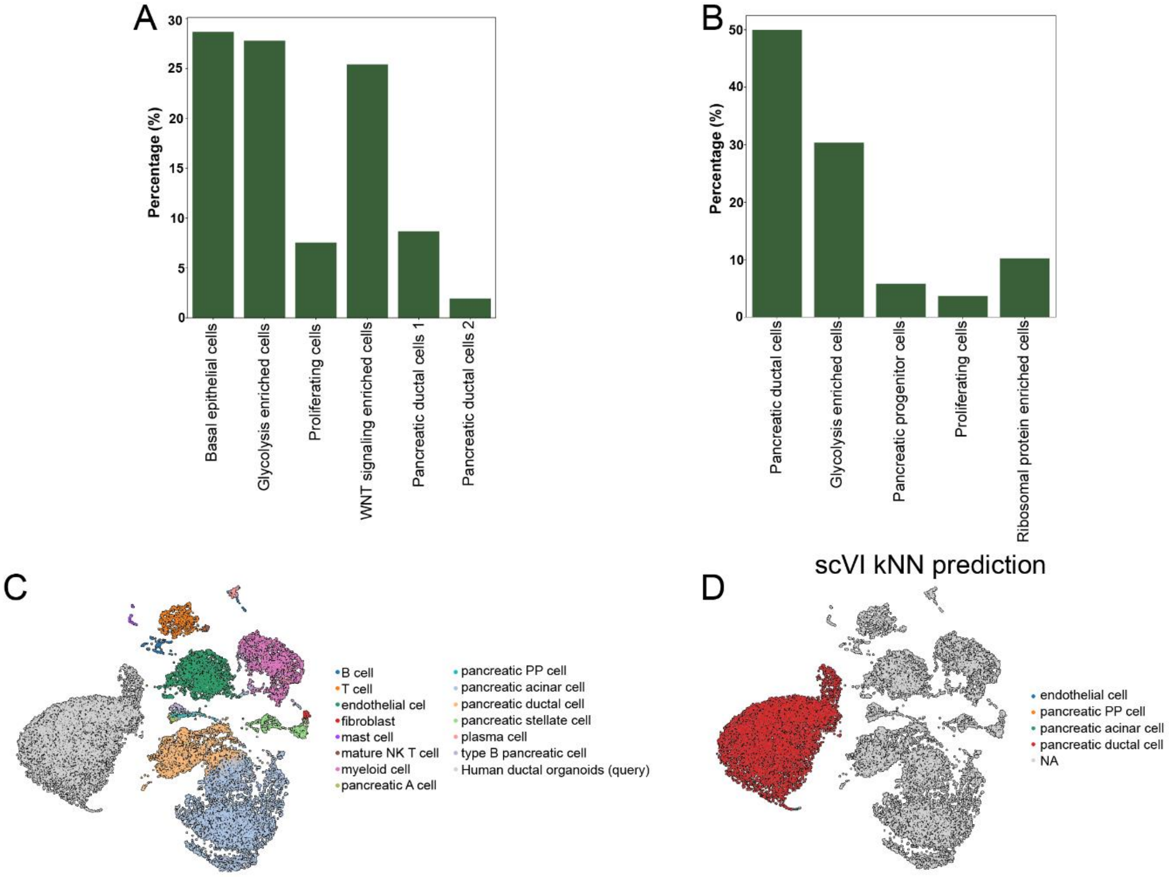

Appendix Figure S1: Cell type/states classification of PPDO/HPDO.
(A-B) Bar-plots showing the percentage of each cell state/type of the integrated scRNA-Seq (A) as well as in HPDO (B).
(C-D) UMAP representation of the cell label transfer of the HPDO form the human tabula sapiens pancreas dataset.
(C) depicts all the cell types of the atlas together with the query cells and (D) is the predicted cell labels to the query dataset using the scVI model.

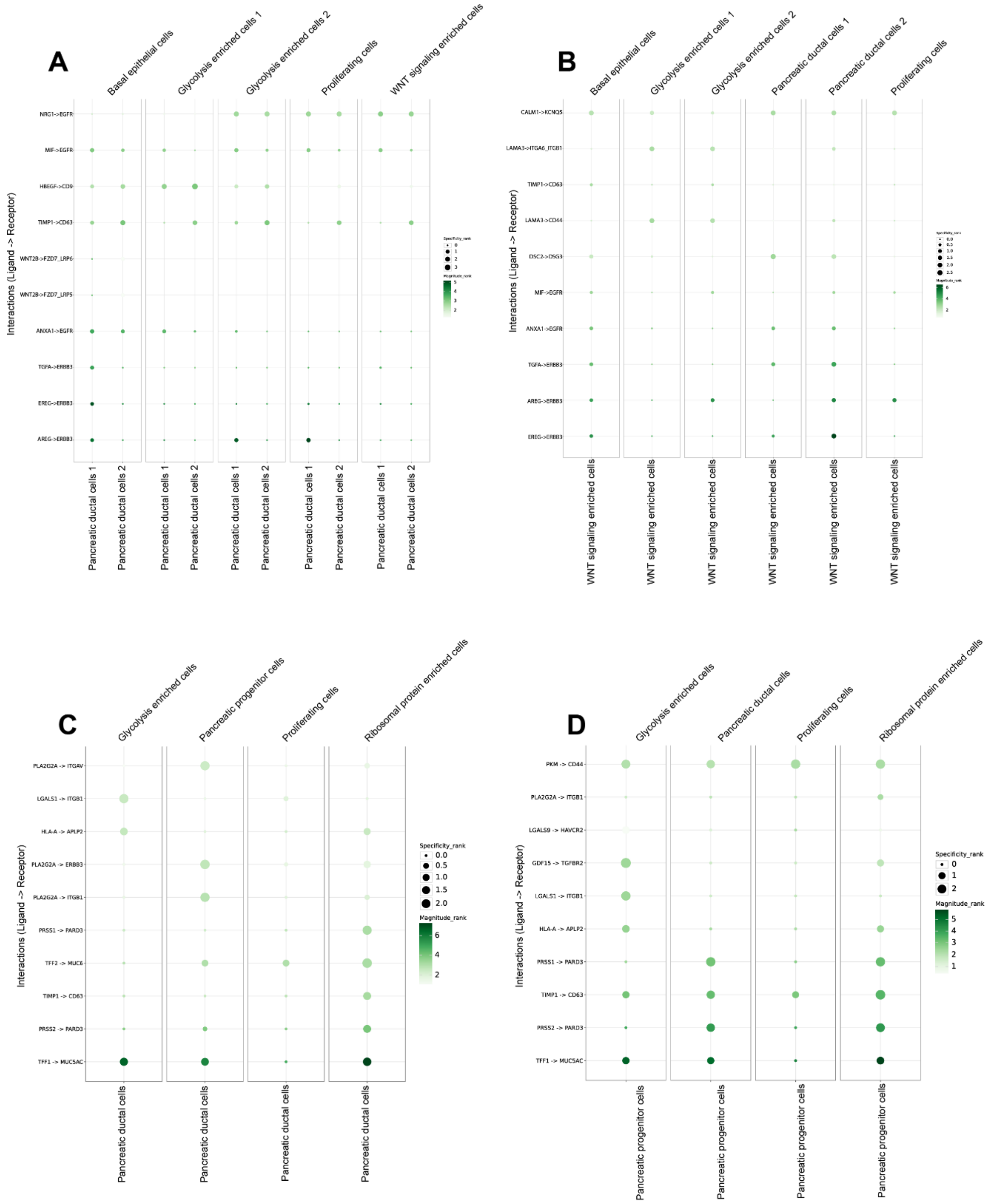

Appendix Figure S2: Ligand-receptor interactions in PPDO/HPDO.
(A-D) Dot plots showing the most significant enriched ligand-receptor interactions towards ductal (A) and progenitor cells (B) in PPDO and (C-D) HPDO scRNA-Seq data respectively.

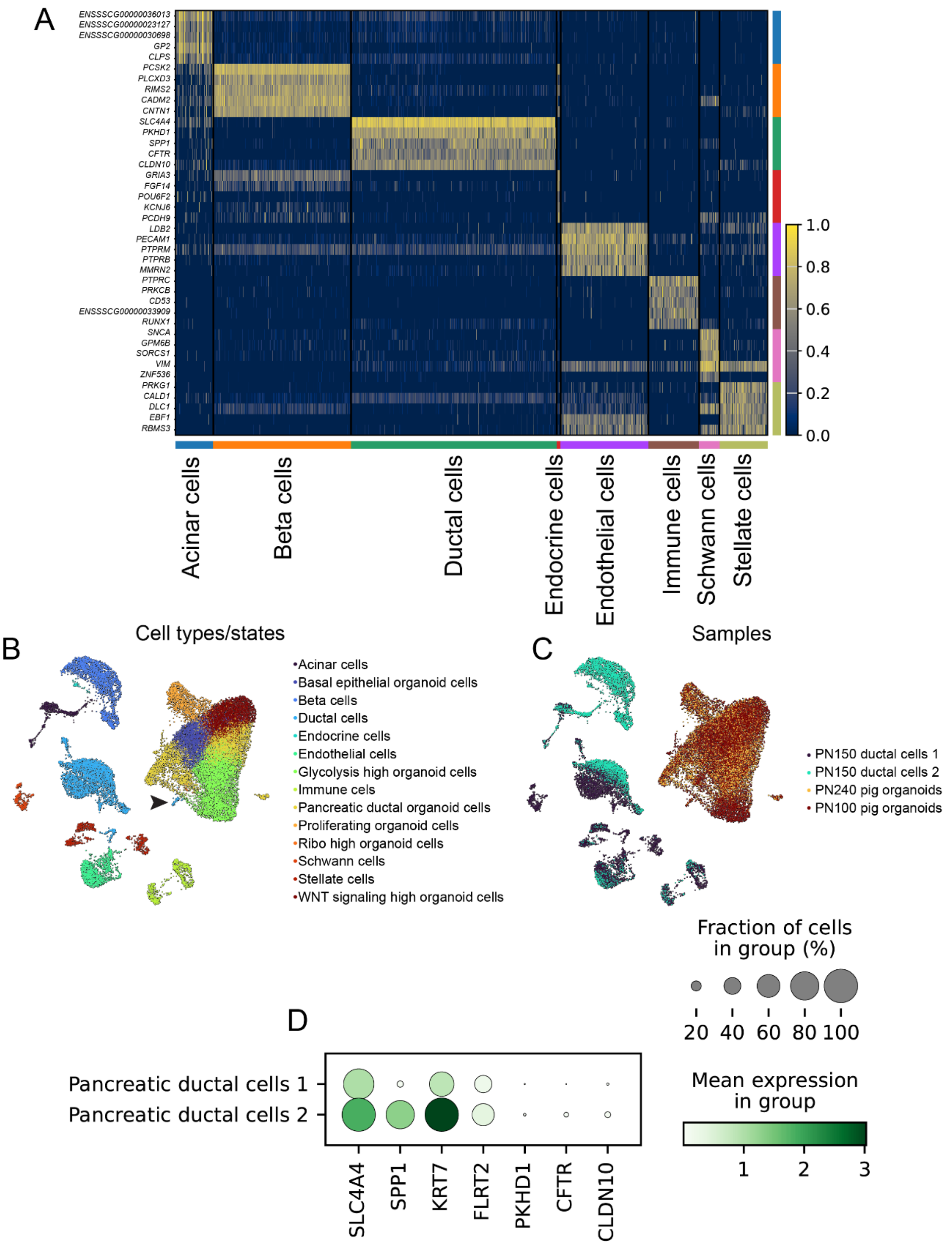

Appendix Figure S3: Characterization of primary porcine dataset
(A) Heatmap showing the top 5 marker genes per cell type identified in the scRNA-Seq data of 2 LPN porcine pancreata.
(B-C) UMAP representation of the integrated PPDO and primary porcine pig pancreas scRNA-Seq datasets with the corresponding annotated cell types/states (B) as well as the corresponding individual samples used for integration (C). Scanorama integration is shown. Black arrowheads point to the PPDO cells classified as primary ductal cells.
(D) Dot plot showing the expression of primary pancreas marker gene of the pancreatic ductal cells in the pancreatic ductal cell 1 and pancreatic ductal cell 2 PPDO dataset.
