# Supplementary figures and images for: Benchmarking porcine pancreatic ductal organoids for drug screening applications
Source: EMBO Mol Med. 2025 Nov 4;17(12):3657–88. doi: 10.1038/s44321-025-00330-3 (PMC12686538; doi:10.1038/s44321-025-00330-3)

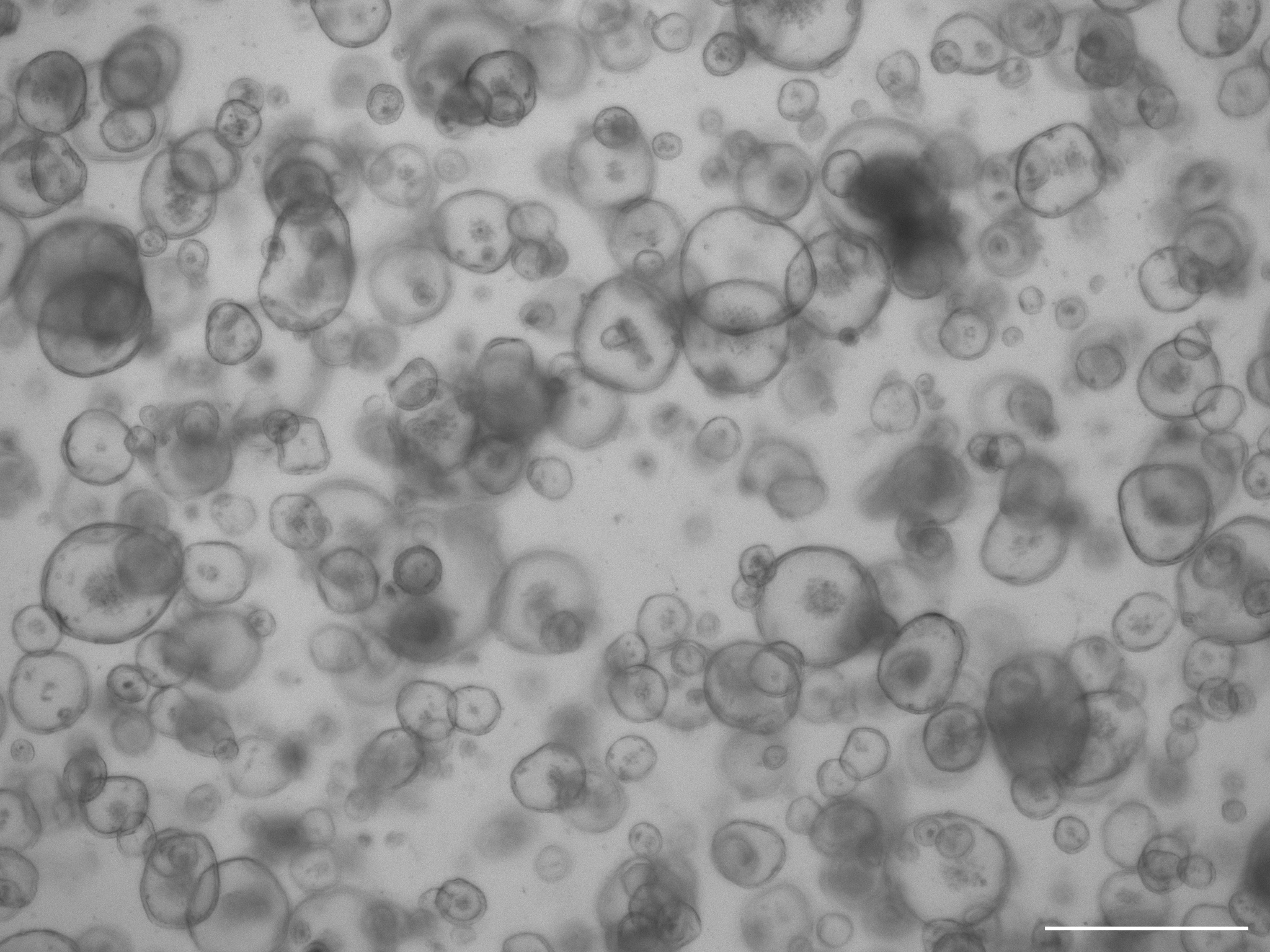

Supplement: Supplementary file 6 — Source data Fig. 1 [file 44321_2025_330_MOESM6_ESM.zip › Figure 1/1B/Brightfield microscopy picture early passage.tif]

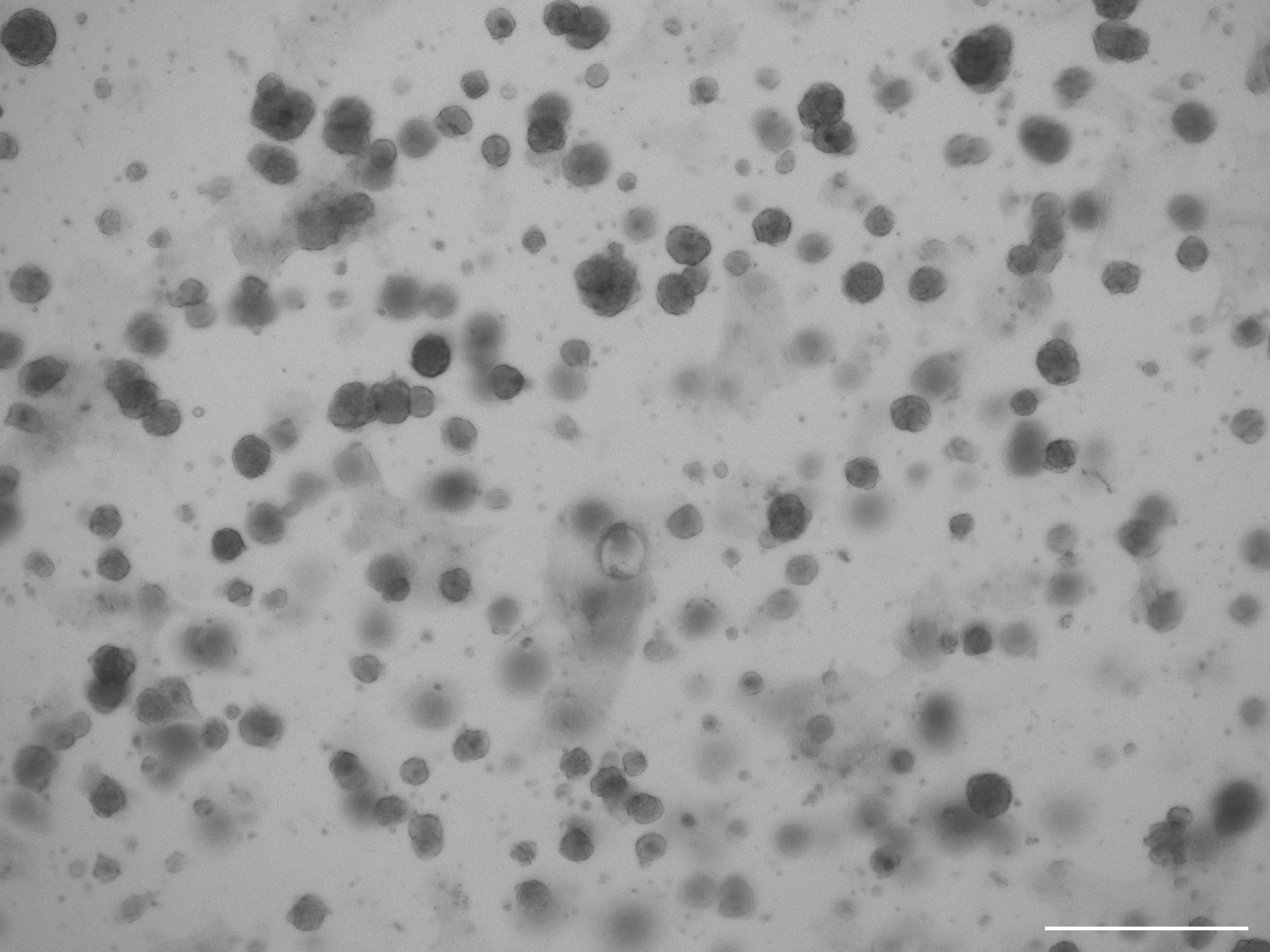

Supplement: Supplementary file 6 — Source data Fig. 1 [file 44321_2025_330_MOESM6_ESM.zip › Figure 1/1B/Brightfield microscopy picture late passage.tif]

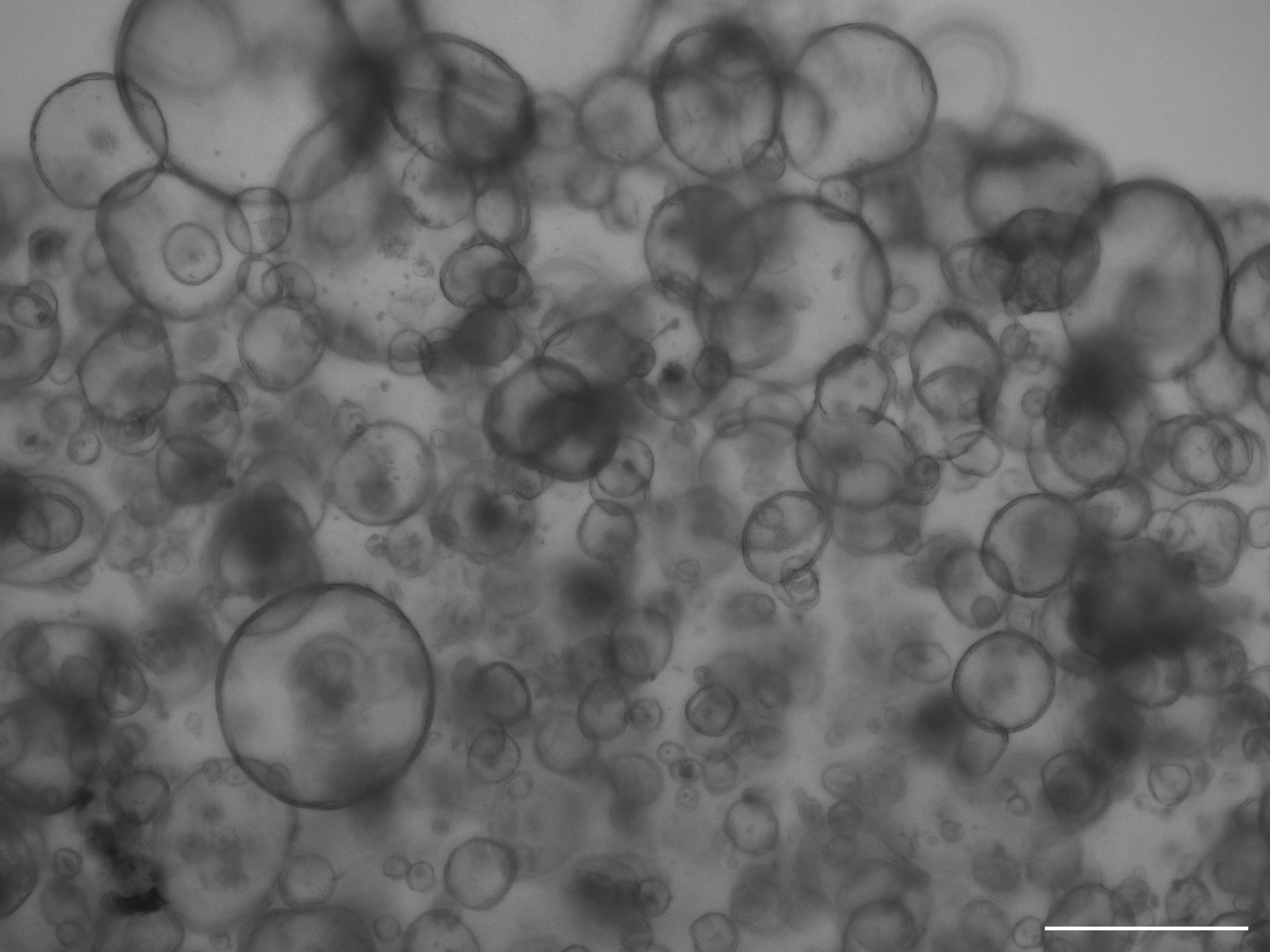

Supplement: Supplementary file 6 — Source data Fig. 1 [file 44321_2025_330_MOESM6_ESM.zip › Figure 1/1C/Brightfield microscopy picture early passage.tif]

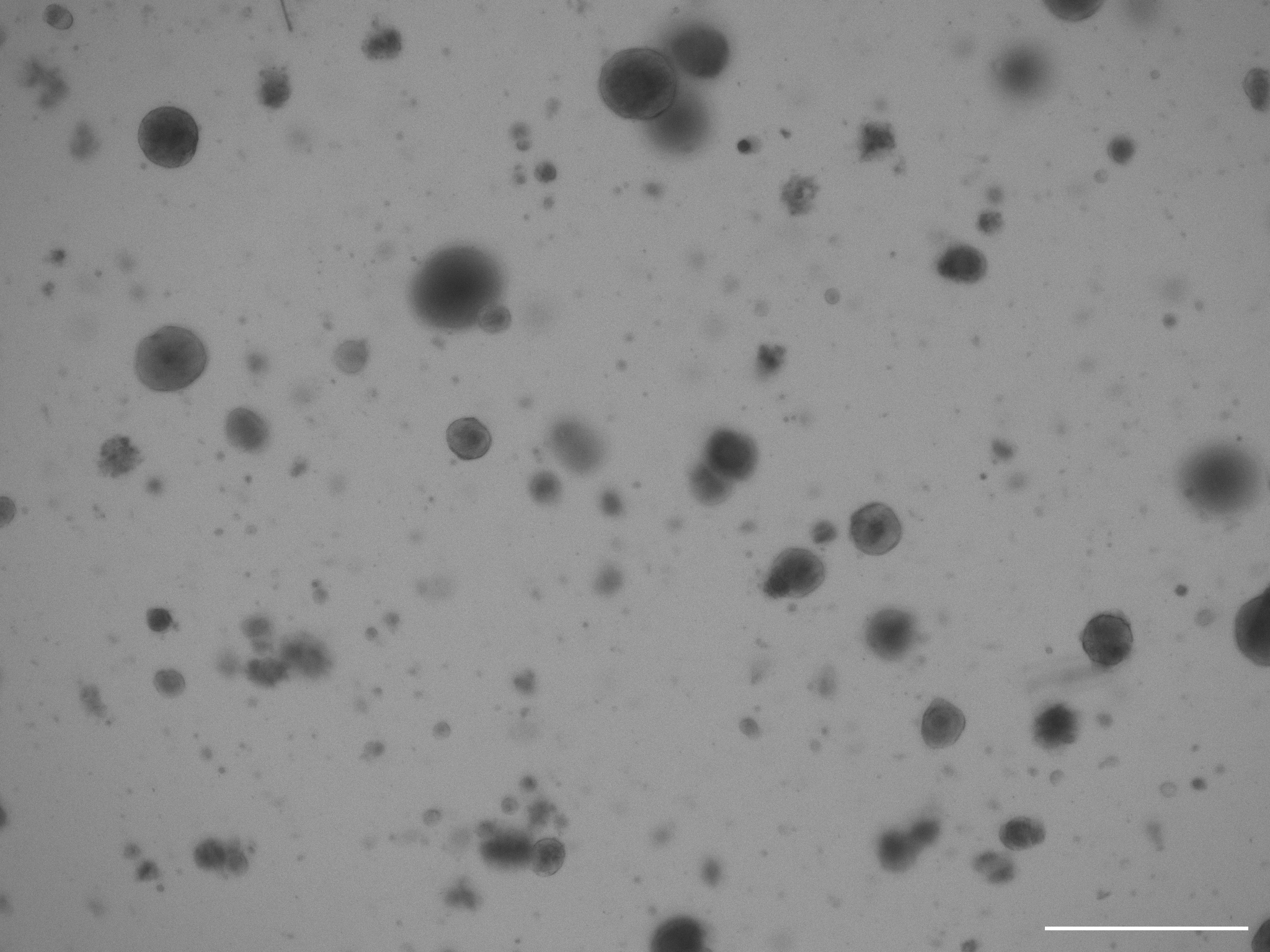

Supplement: Supplementary file 6 — Source data Fig. 1 [file 44321_2025_330_MOESM6_ESM.zip › Figure 1/1C/Brightfield microscopy picture Late passage.tif]

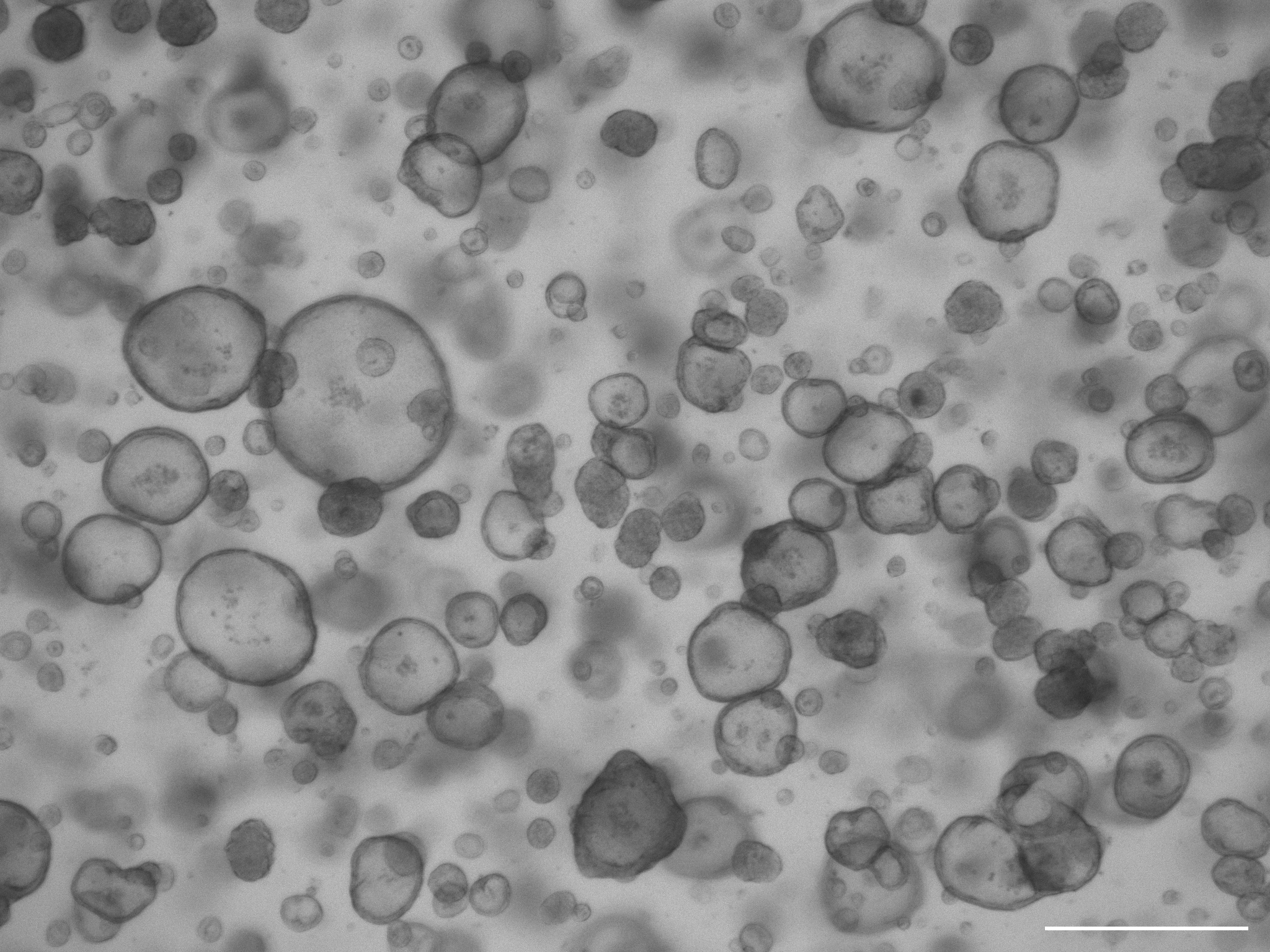

Supplement: Supplementary file 6 — Source data Fig. 1 [file 44321_2025_330_MOESM6_ESM.zip › Figure 1/1D/Brightfield microscopy picture early passage.tif]

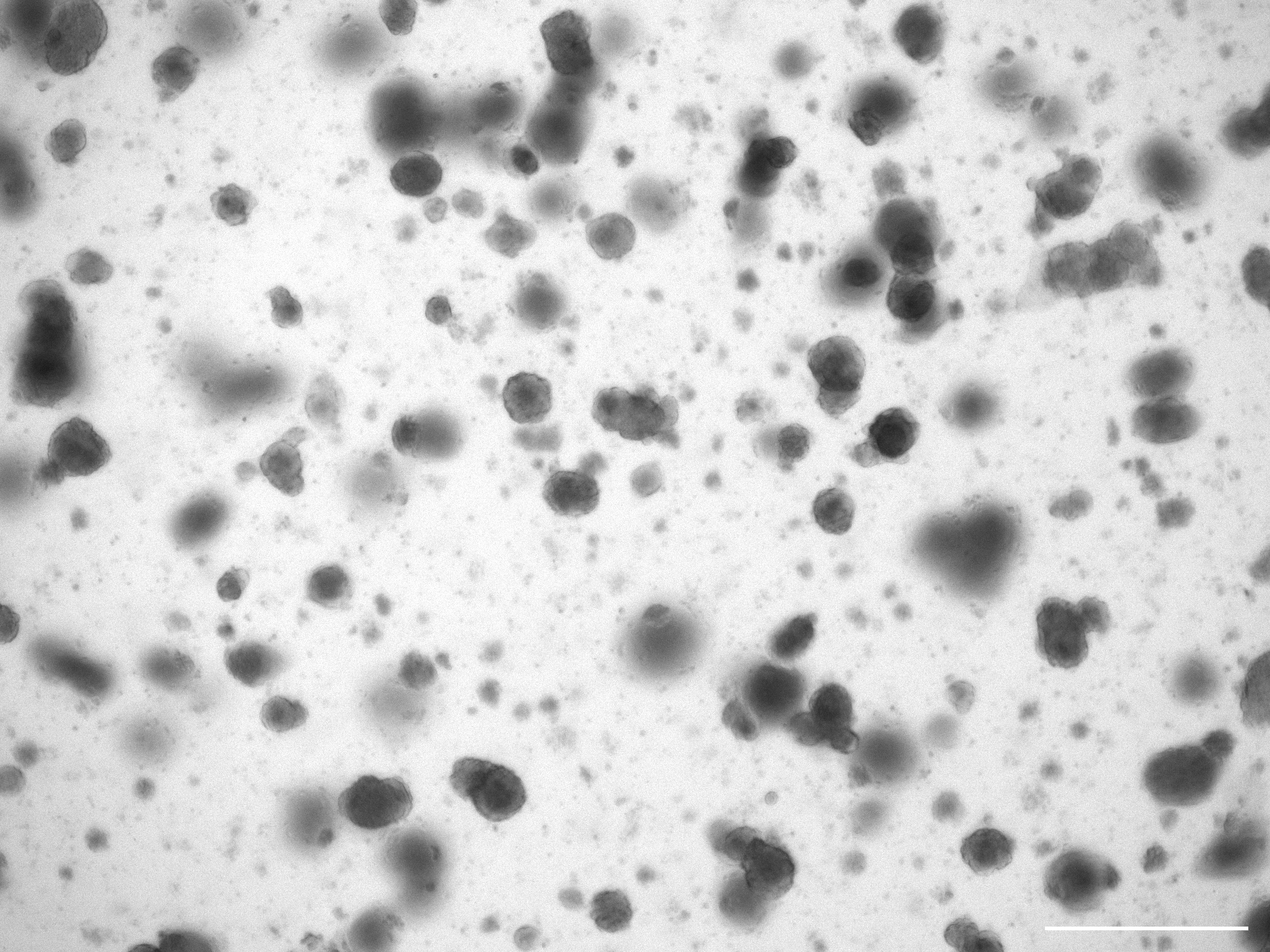

Supplement: Supplementary file 6 — Source data Fig. 1 [file 44321_2025_330_MOESM6_ESM.zip › Figure 1/1D/Brightfield microscopy picture Late passage.tif]

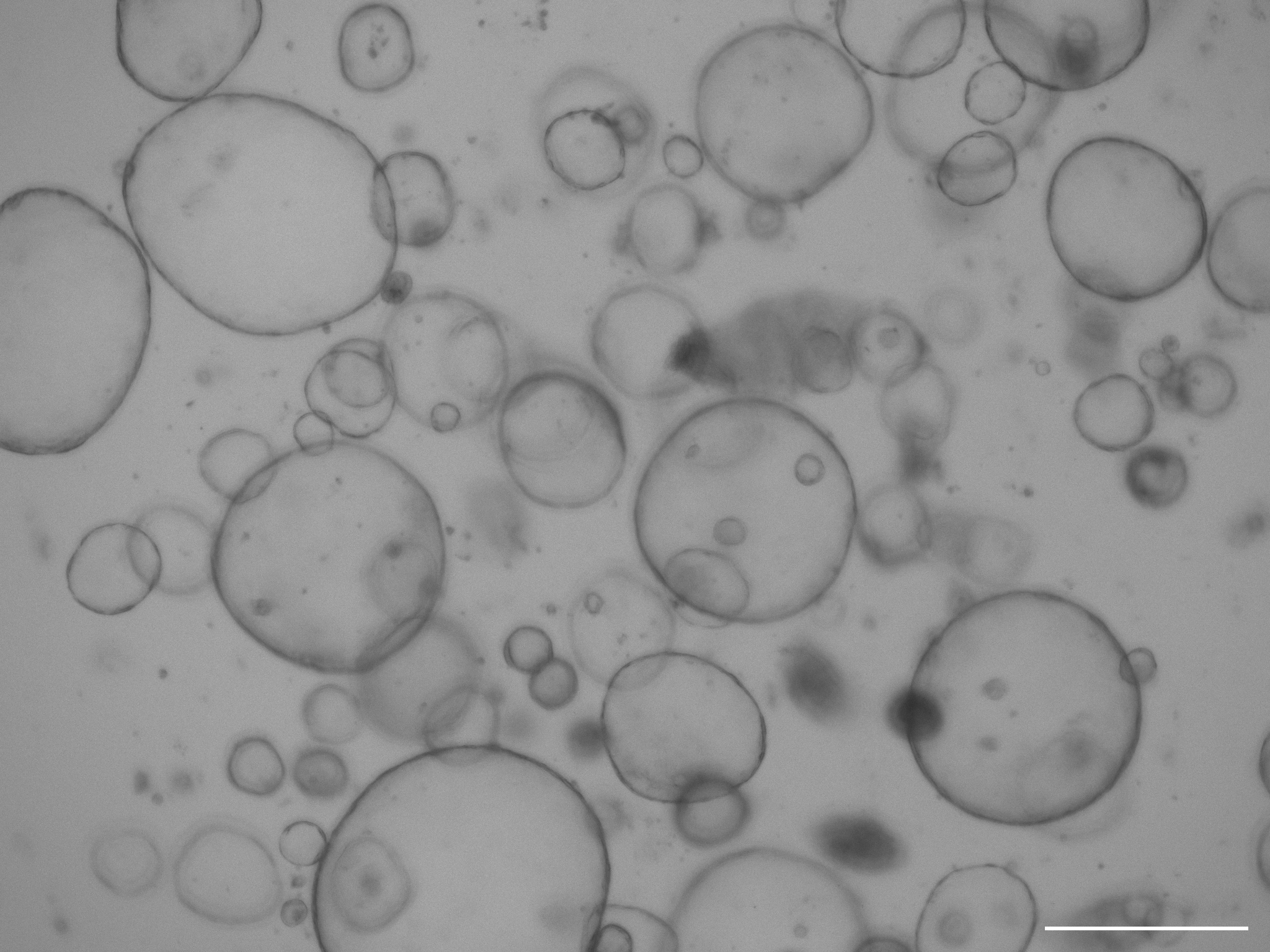

Supplement: Supplementary file 6 — Source data Fig. 1 [file 44321_2025_330_MOESM6_ESM.zip › Figure 1/1E/Brightfield microscopy picture early passage.tiff]

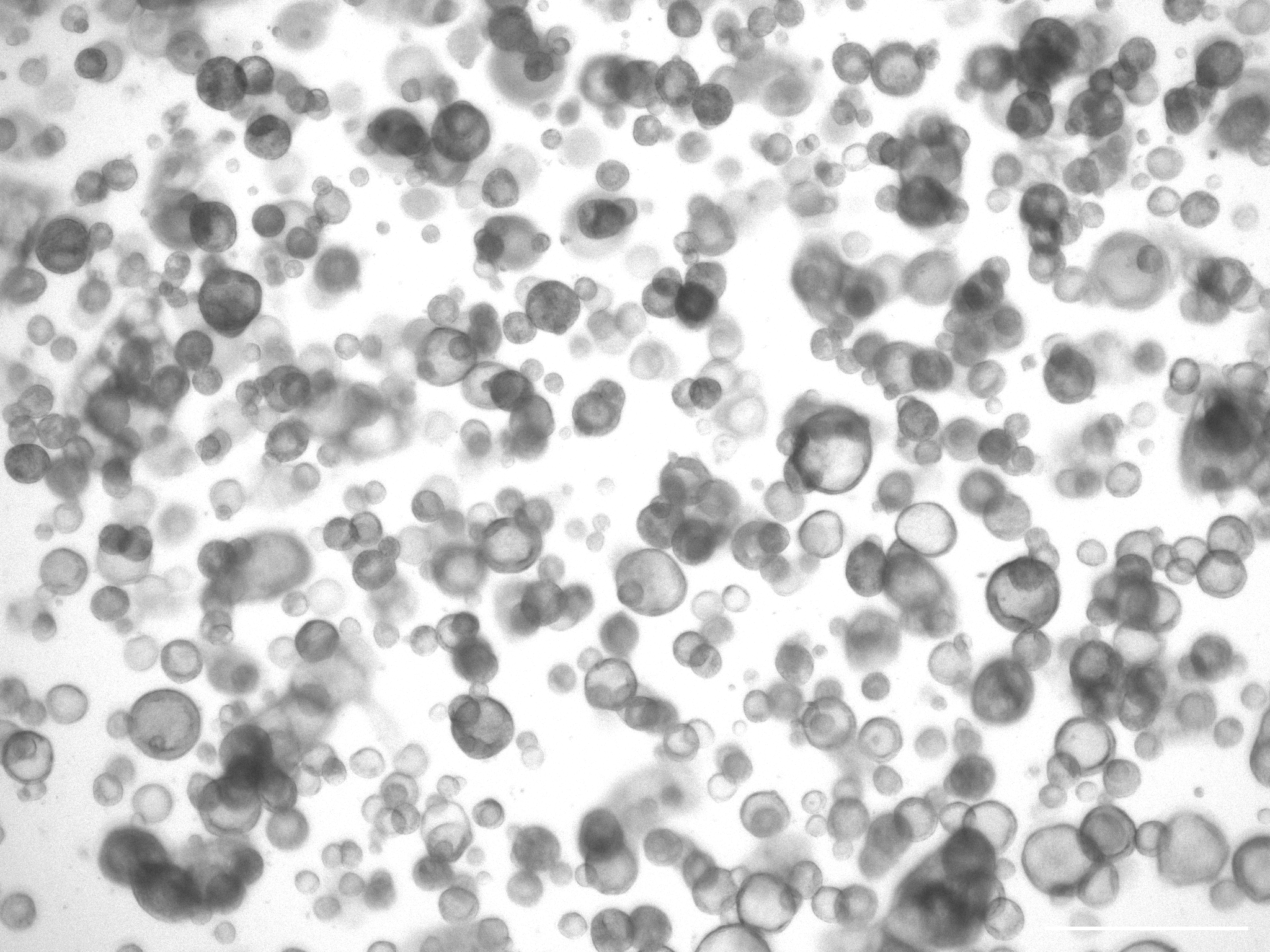

Supplement: Supplementary file 6 — Source data Fig. 1 [file 44321_2025_330_MOESM6_ESM.zip › Figure 1/1E/Brightfield microscopy picture Late passage.tiff]

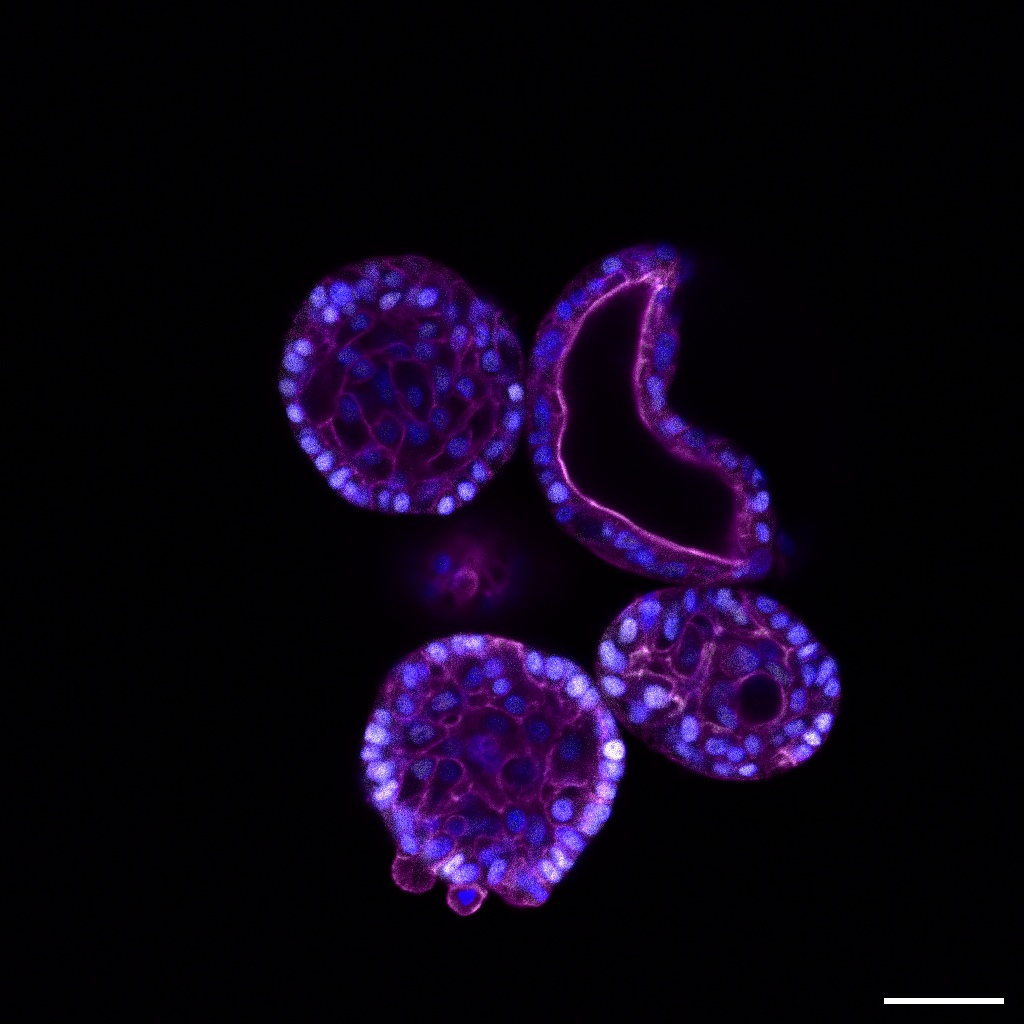

Supplement: Supplementary file 6 — Source data Fig. 1 [file 44321_2025_330_MOESM6_ESM.zip › Figure 1/1F/Merge confocal microscopy picture single plane.tif]

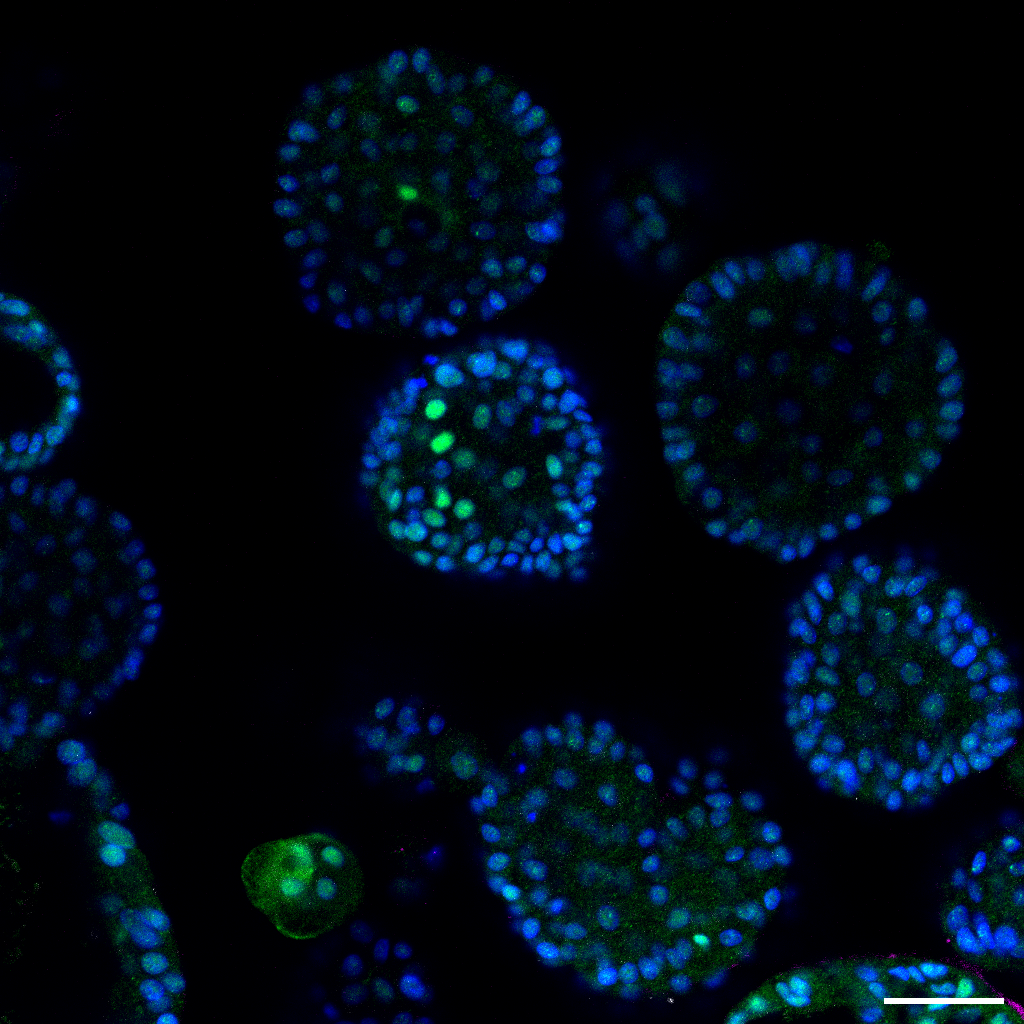

Supplement: Supplementary file 6 — Source data Fig. 1 [file 44321_2025_330_MOESM6_ESM.zip › Figure 1/1G/Merge confocal microscopy picture single plane.tif]

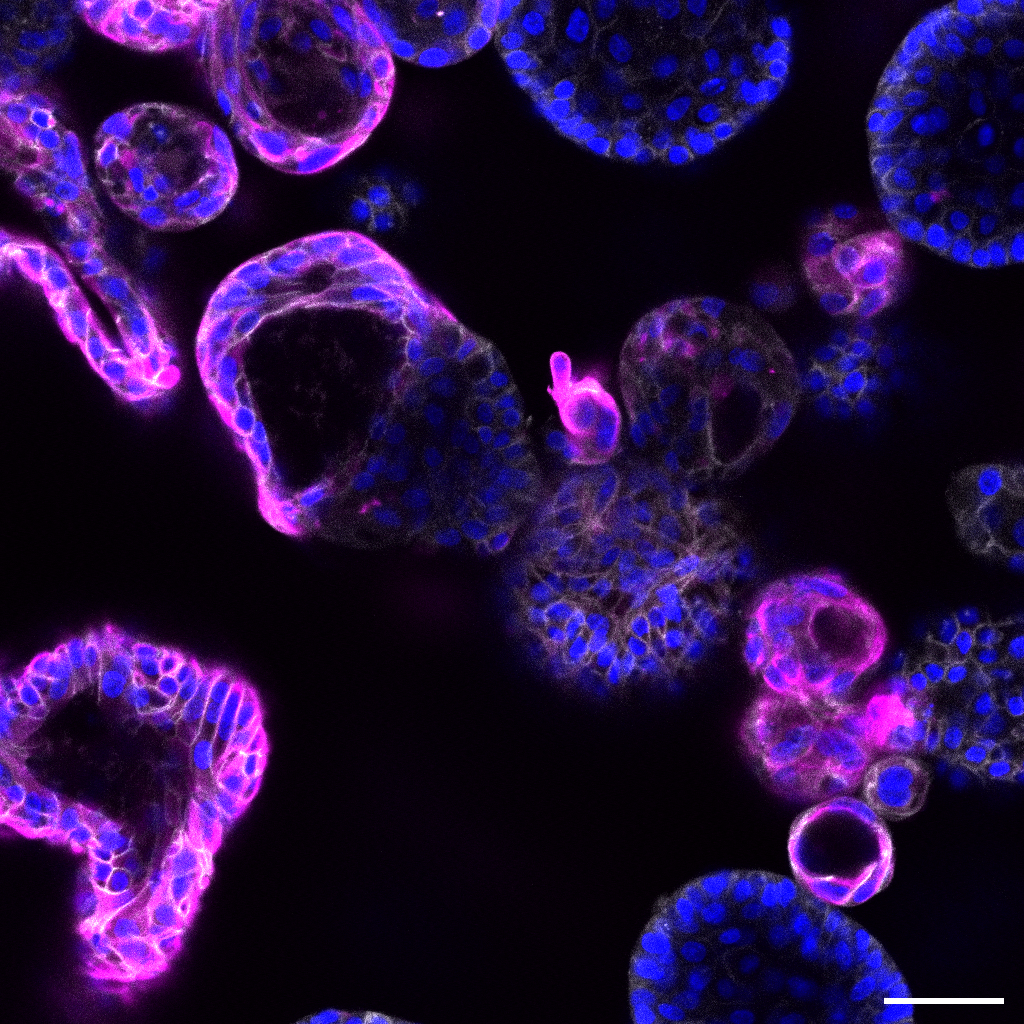

Supplement: Supplementary file 6 — Source data Fig. 1 [file 44321_2025_330_MOESM6_ESM.zip › Figure 1/1H/Merge confocal microscopy picture single plane.tif]

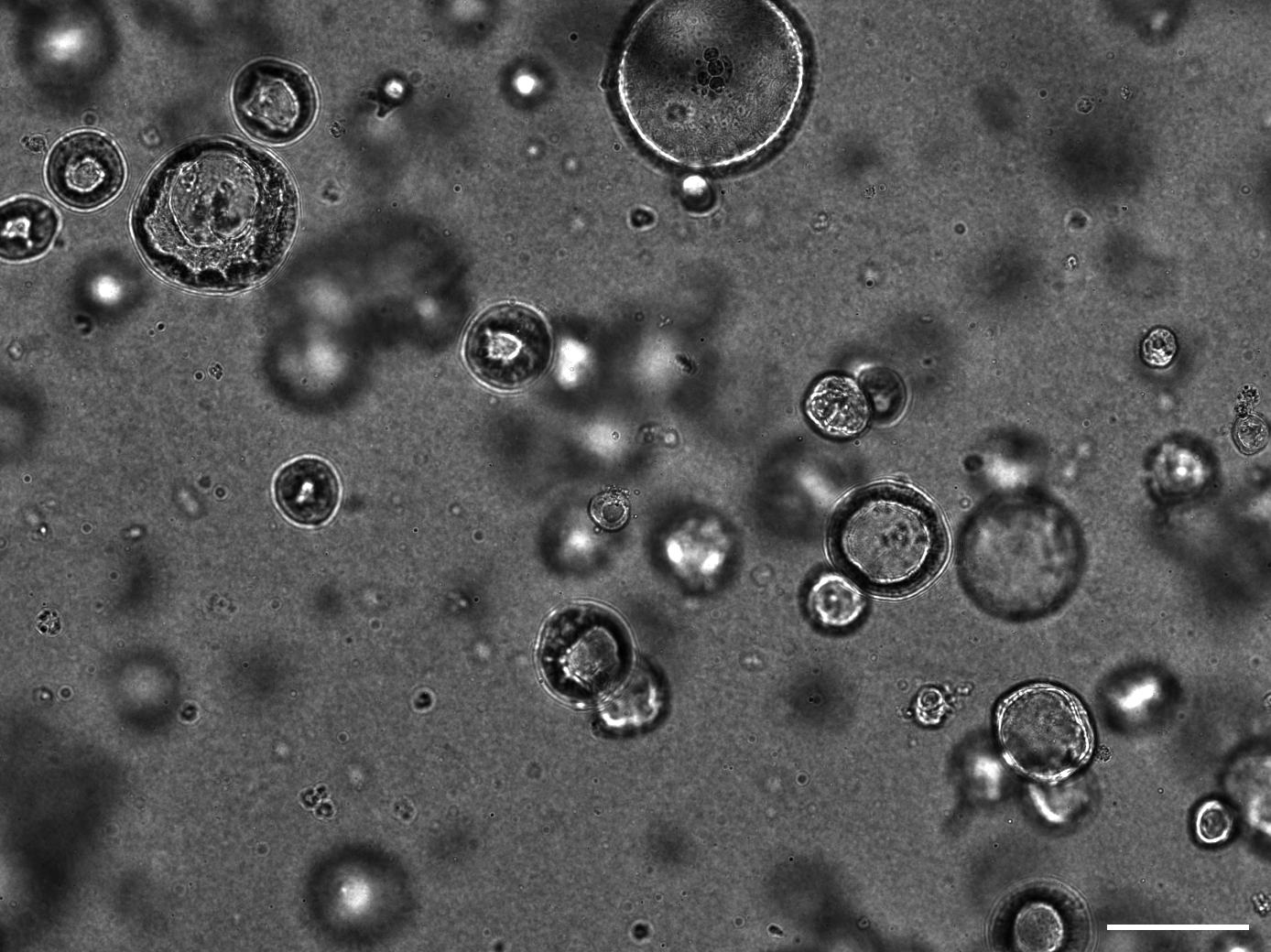

Supplement: Supplementary file 6 — Source data Fig. 1 [file 44321_2025_330_MOESM6_ESM.zip › Figure 1/1I/Brightfield microscopy picture DMSO 0hr.tif]

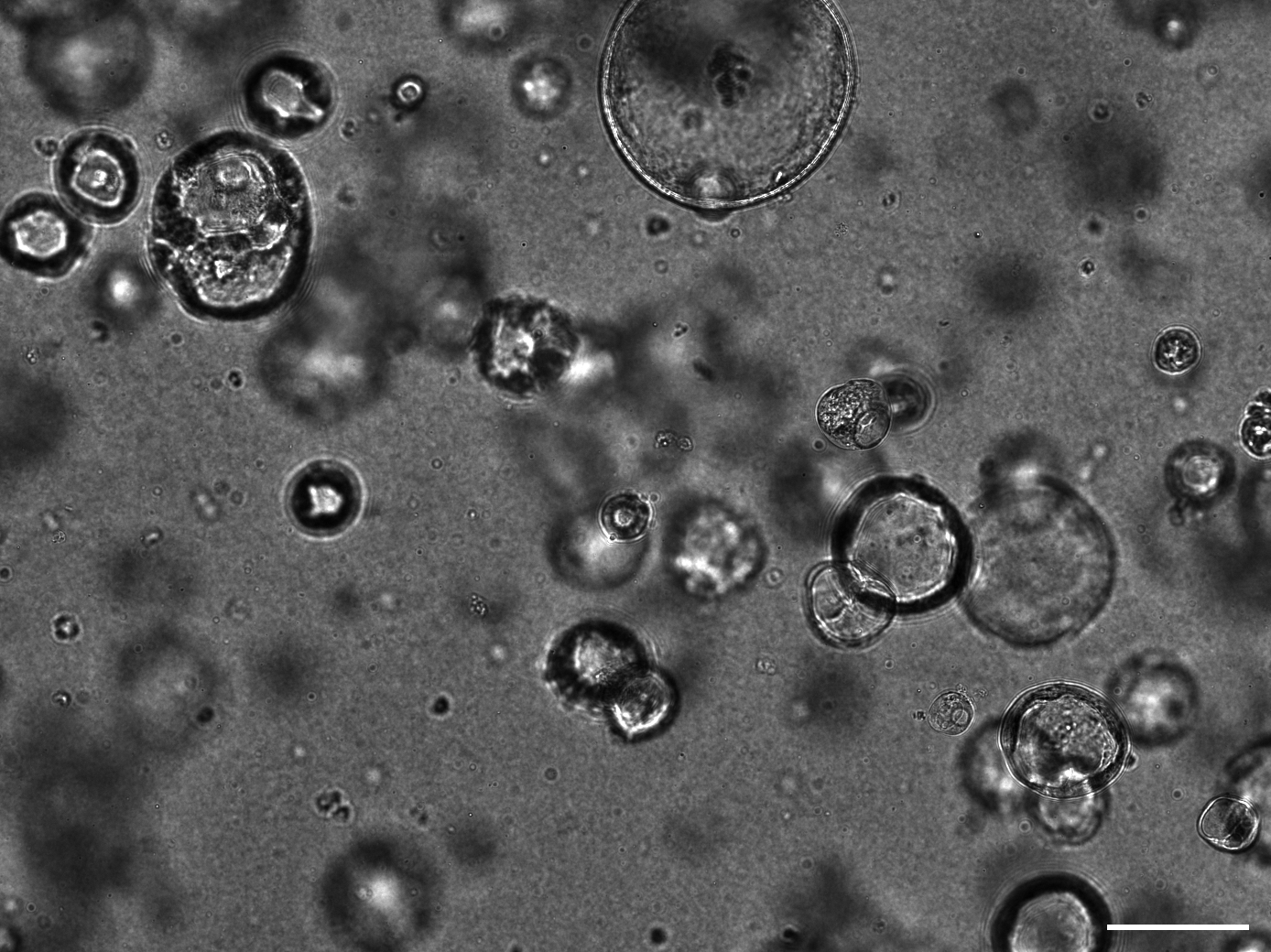

Supplement: Supplementary file 6 — Source data Fig. 1 [file 44321_2025_330_MOESM6_ESM.zip › Figure 1/1I/Brightfield microscopy picture dmso 4hr.tif]

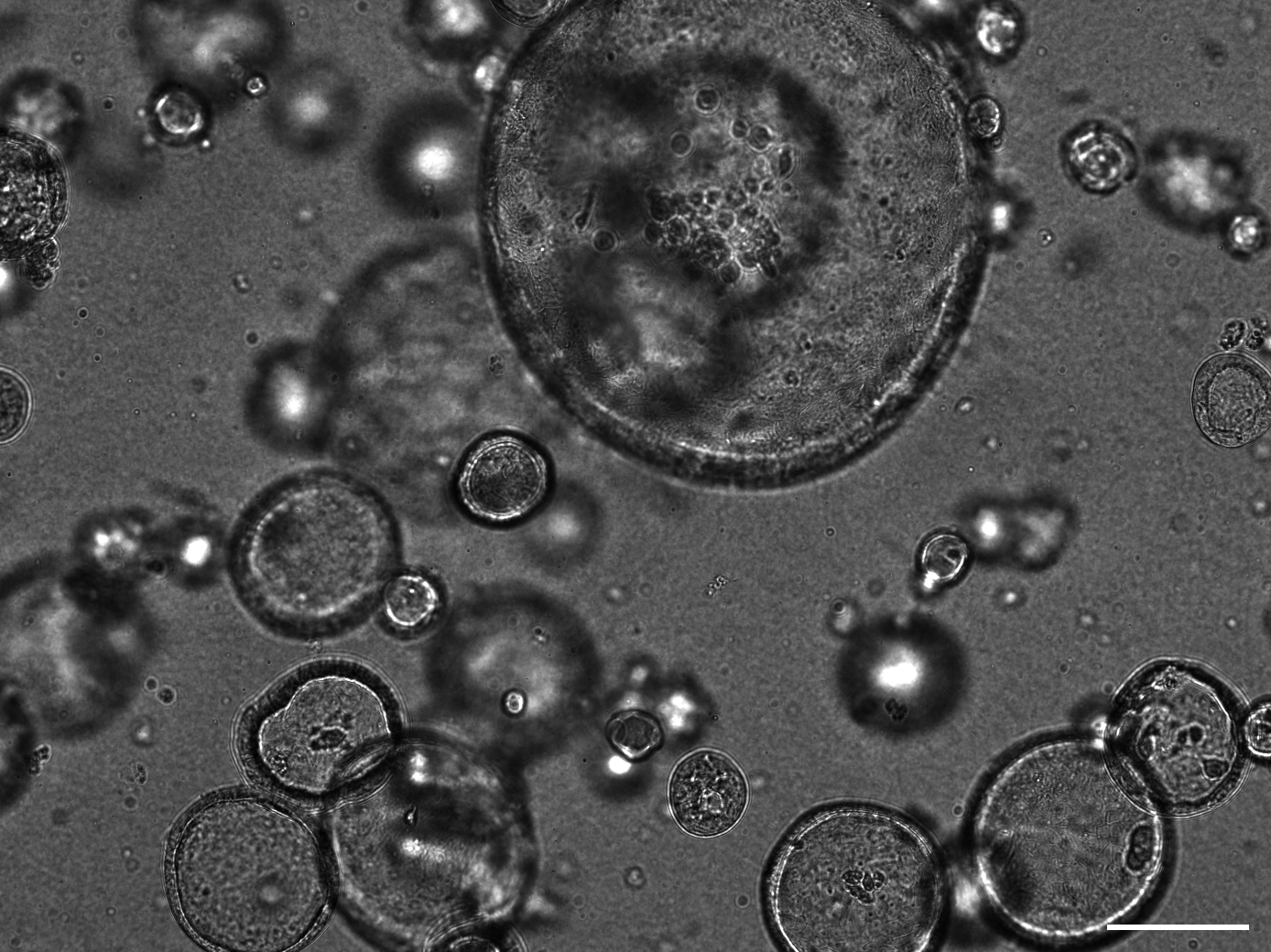

Supplement: Supplementary file 6 — Source data Fig. 1 [file 44321_2025_330_MOESM6_ESM.zip › Figure 1/1J/Brightfield microscopy picture 0 hr forskolin.tif]

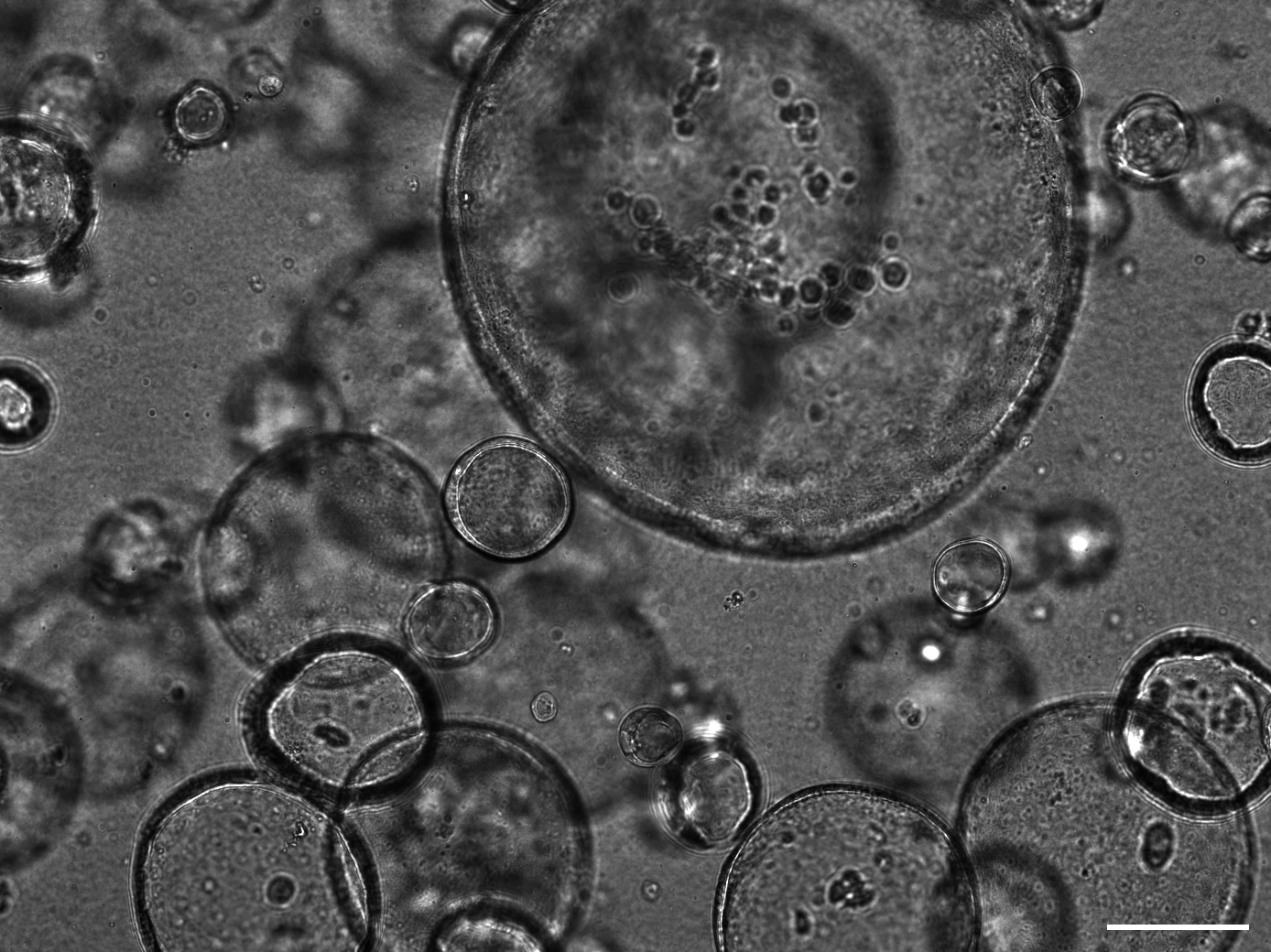

Supplement: Supplementary file 6 — Source data Fig. 1 [file 44321_2025_330_MOESM6_ESM.zip › Figure 1/1J/Brightfield microscopy picture 4 hr forskolin.tif]

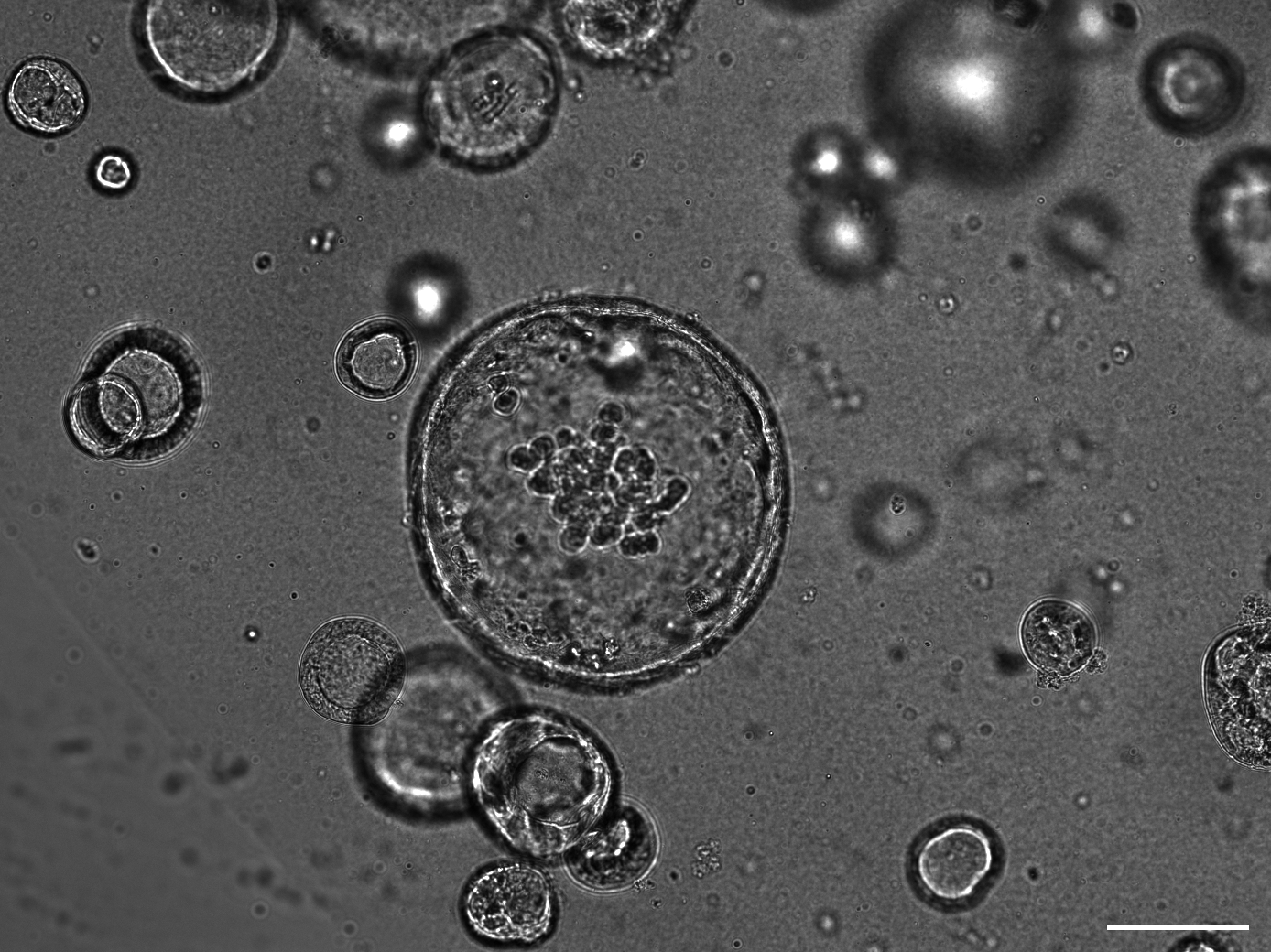

Supplement: Supplementary file 6 — Source data Fig. 1 [file 44321_2025_330_MOESM6_ESM.zip › Figure 1/1K/Brightfield microscopy picture 0hr.tif]

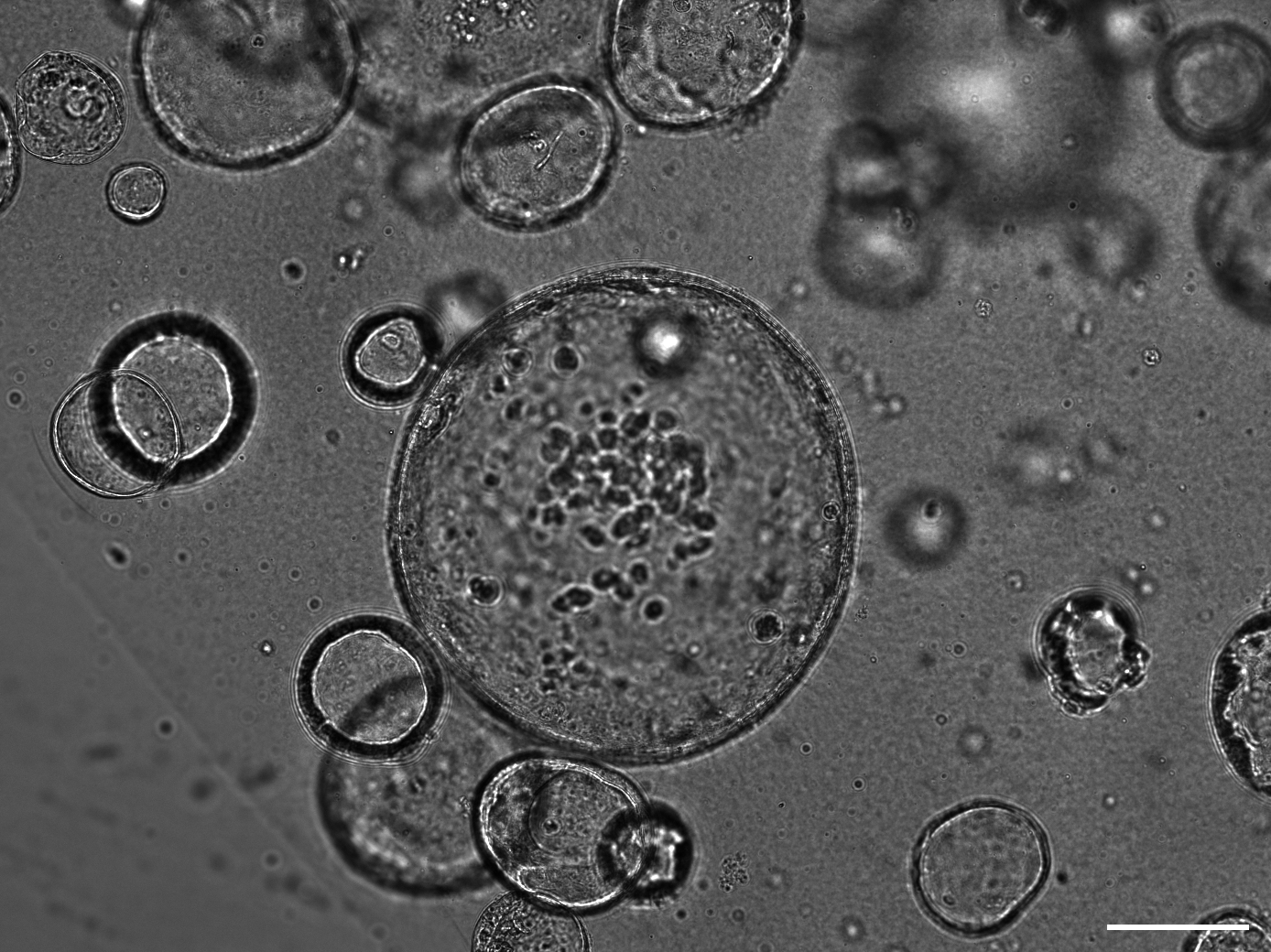

Supplement: Supplementary file 6 — Source data Fig. 1 [file 44321_2025_330_MOESM6_ESM.zip › Figure 1/1K/Brightfield microscopy picture 4hr.tif]

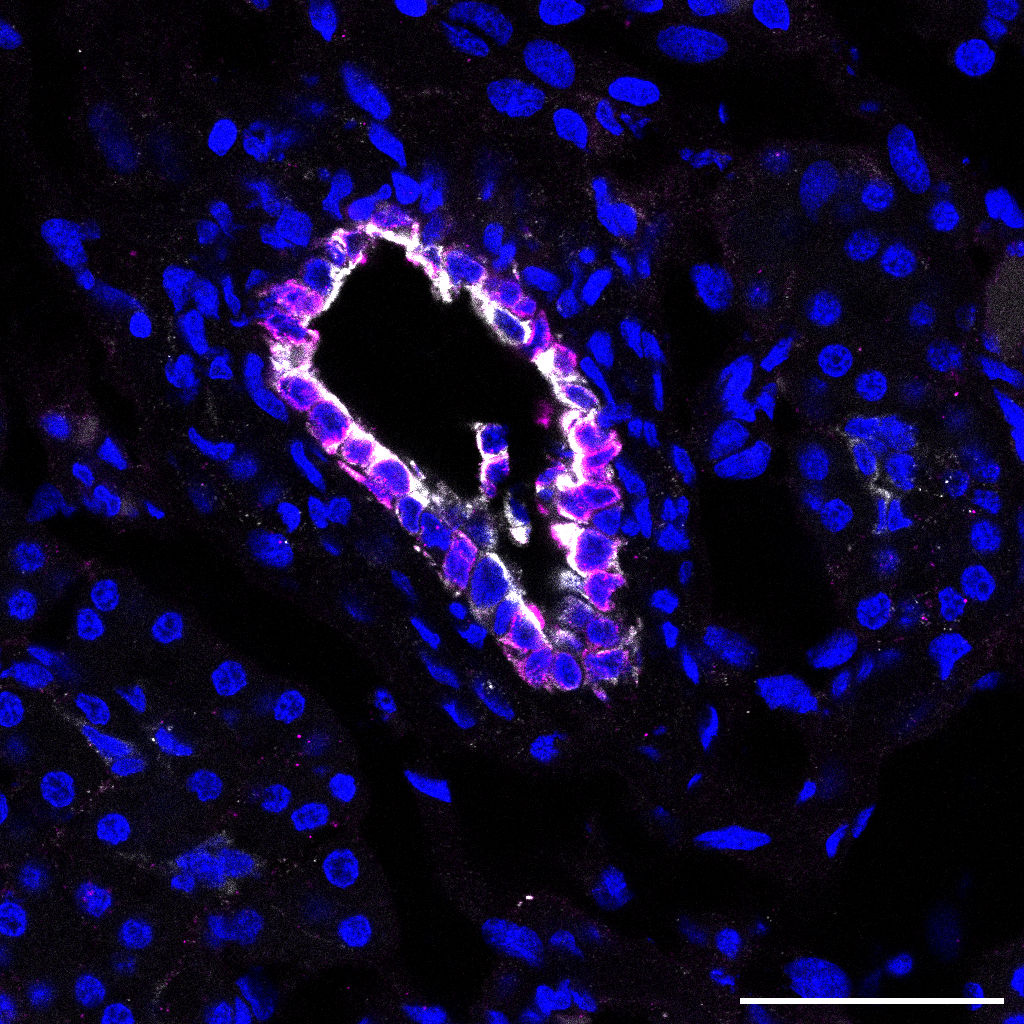

Supplement: Supplementary file 7 — Source data Fig. 4 [file 44321_2025_330_MOESM7_ESM.zip › Figure 4/4F/Confocal microscopy image pig pancreas tissue.tif]

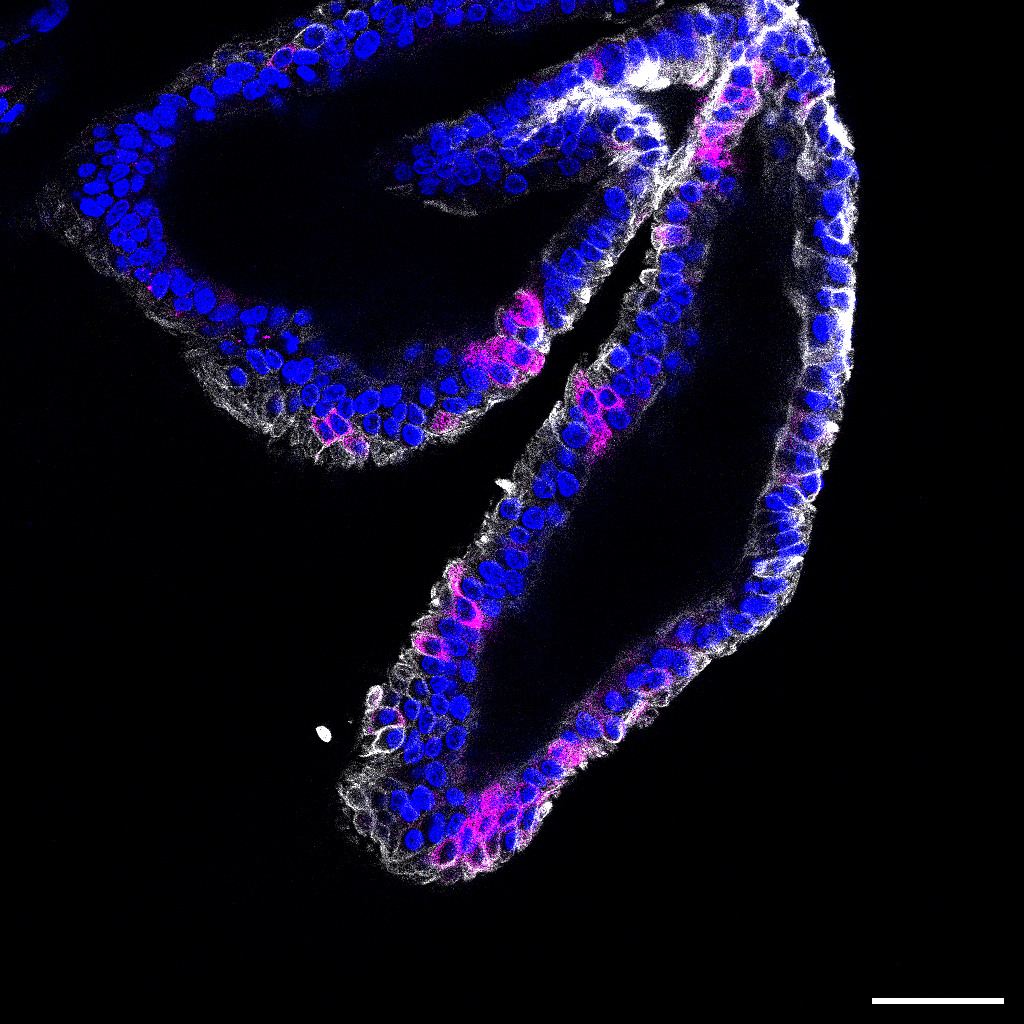

Supplement: Supplementary file 7 — Source data Fig. 4 [file 44321_2025_330_MOESM7_ESM.zip › Figure 4/4G/Confocal microscopy image pig pancreas organoids.tif]

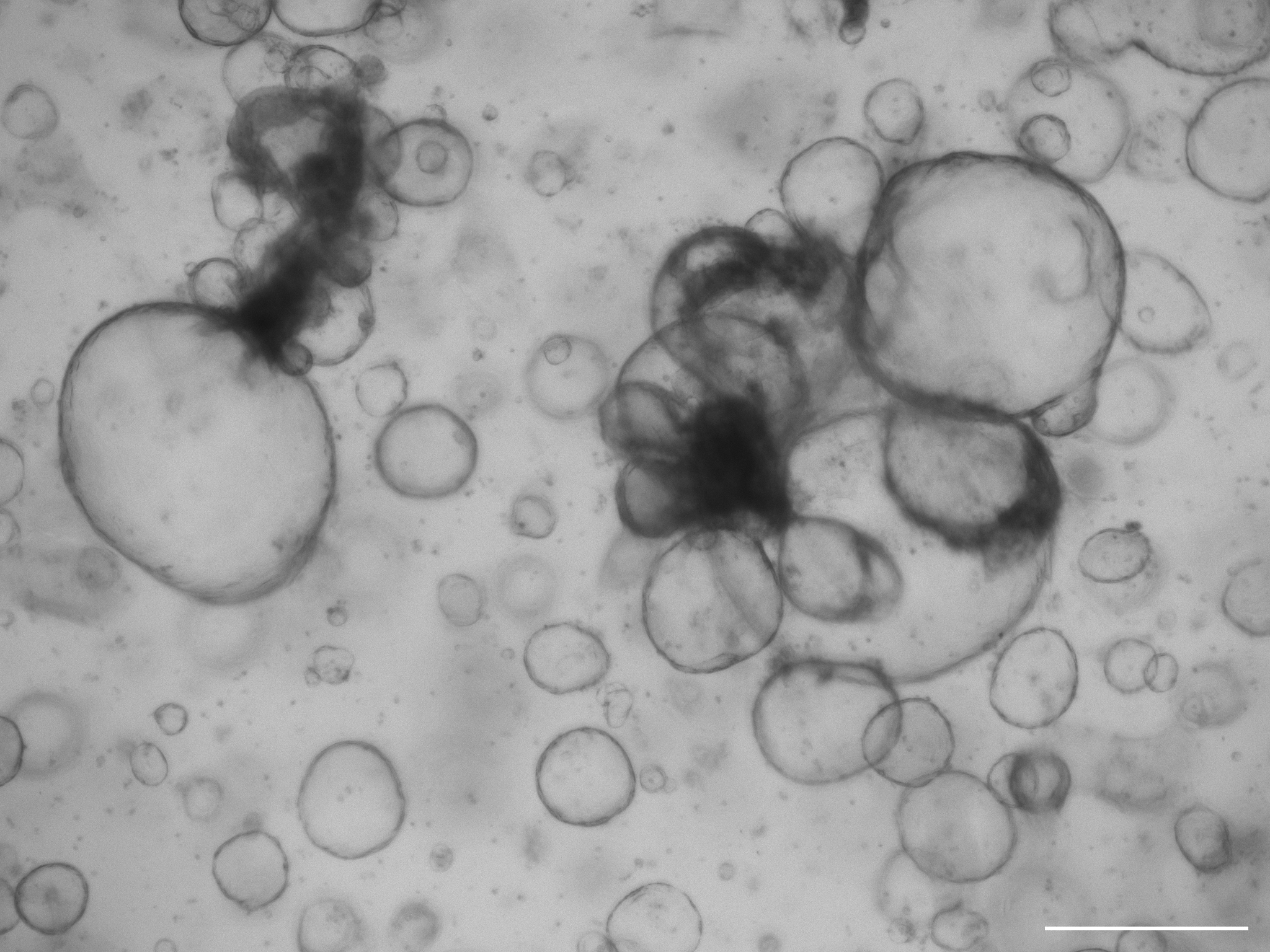

Supplement: Supplementary file 7 — Source data Fig. 4 [file 44321_2025_330_MOESM7_ESM.zip › Figure 4/4H/Brightfield microscopy picture PPDO.tif]

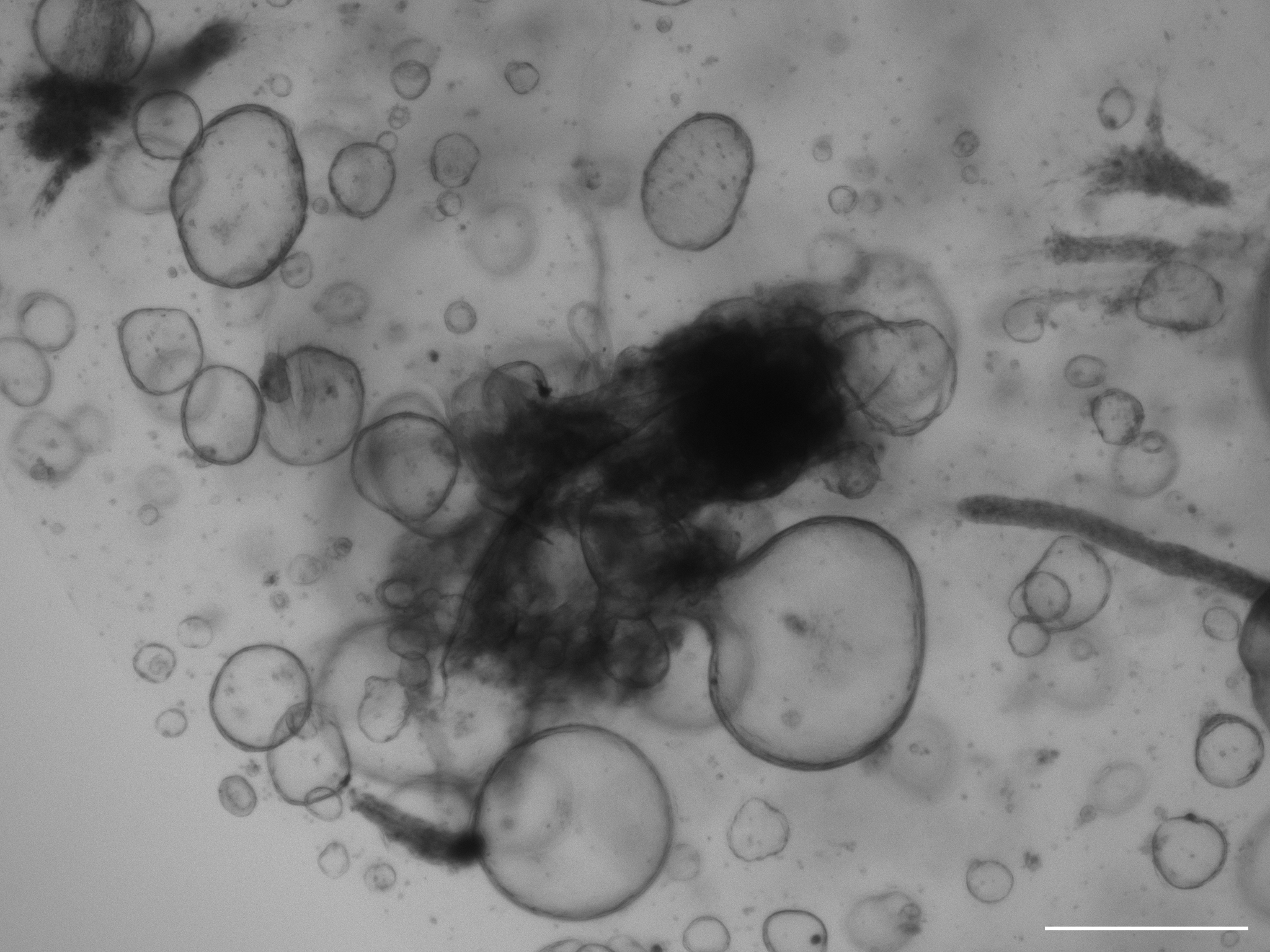

Supplement: Supplementary file 7 — Source data Fig. 4 [file 44321_2025_330_MOESM7_ESM.zip › Figure 4/4I/Brightfield microscopy picture PPDO.tif]

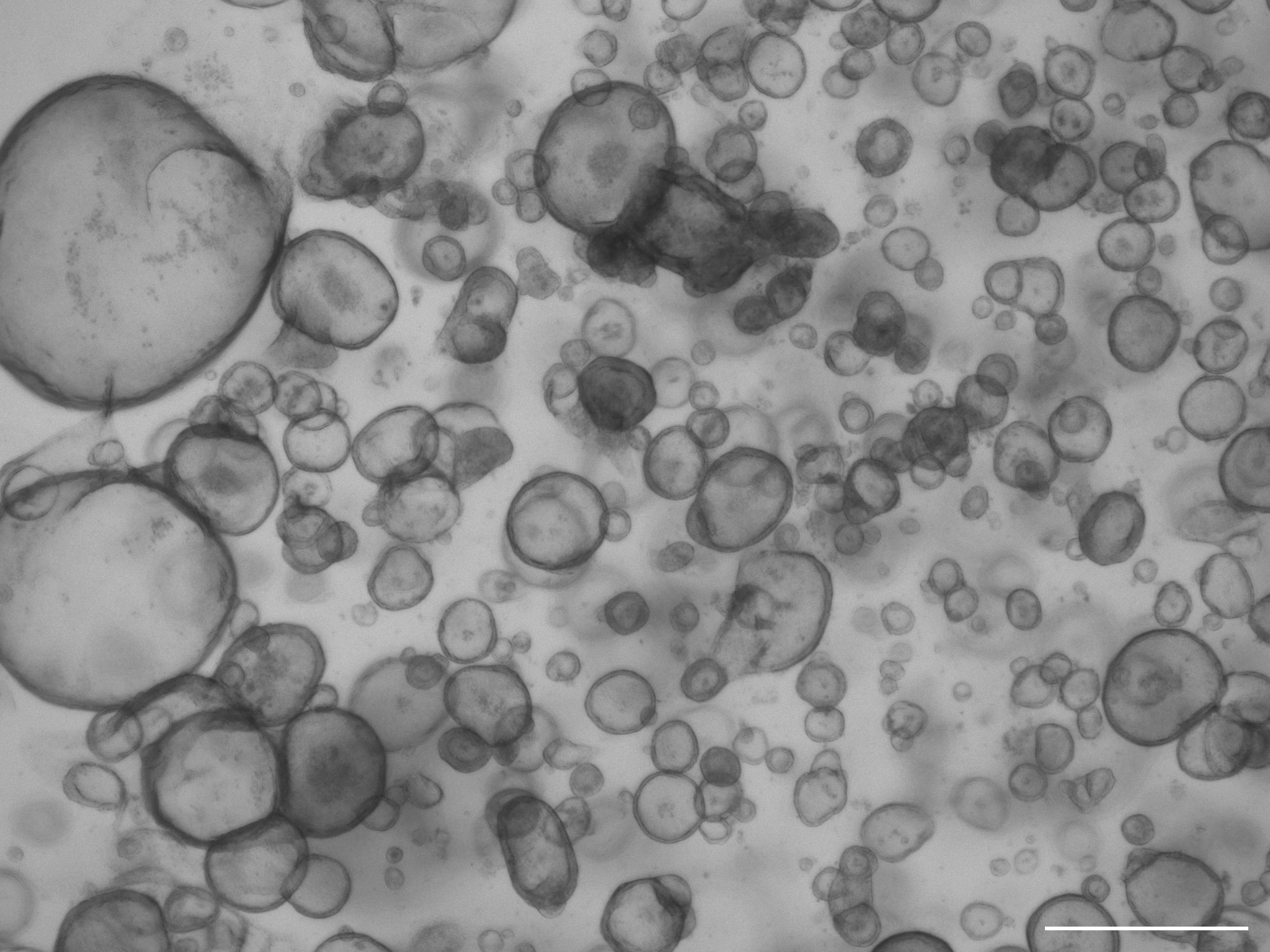

Supplement: Supplementary file 7 — Source data Fig. 4 [file 44321_2025_330_MOESM7_ESM.zip › Figure 4/4J/Brightfield microscopy picture PPDO.tif]

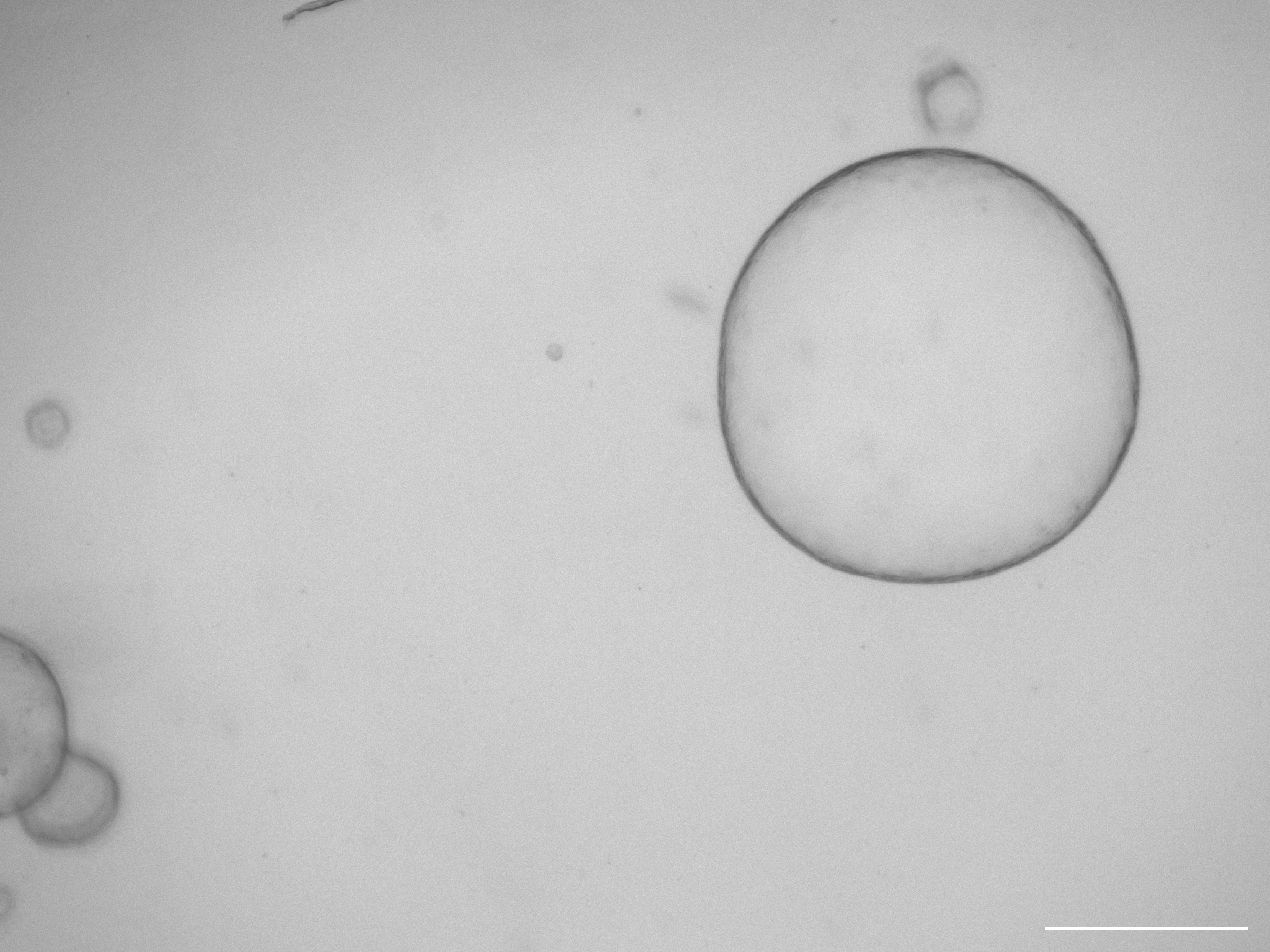

Supplement: Supplementary file 7 — Source data Fig. 4 [file 44321_2025_330_MOESM7_ESM.zip › Figure 4/4K/Brightfield microscopy picture PPDO.tif]

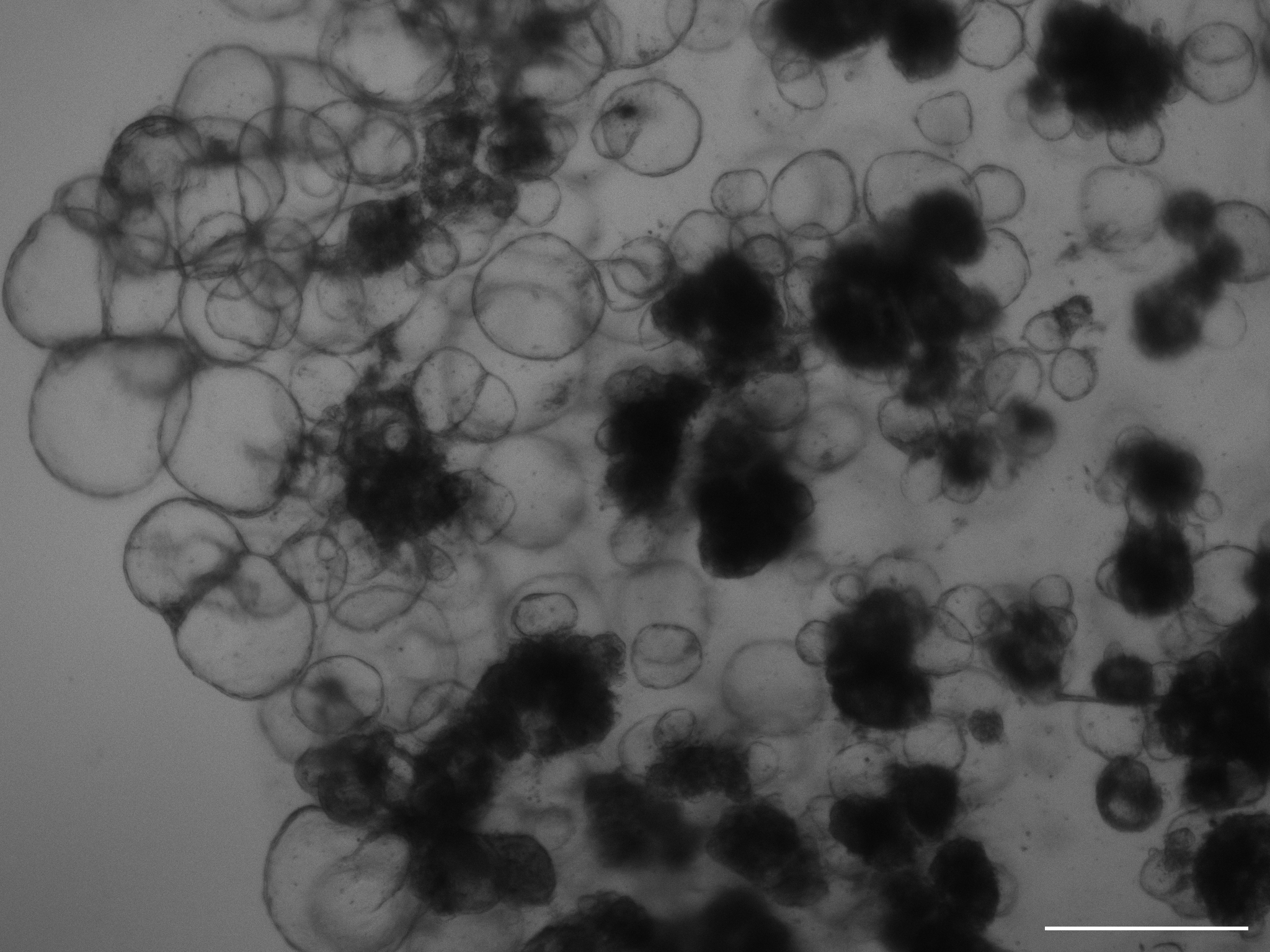

Supplement: Supplementary file 7 — Source data Fig. 4 [file 44321_2025_330_MOESM7_ESM.zip › Figure 4/4L/Brightfield microscopy picture HPDO.tiff]

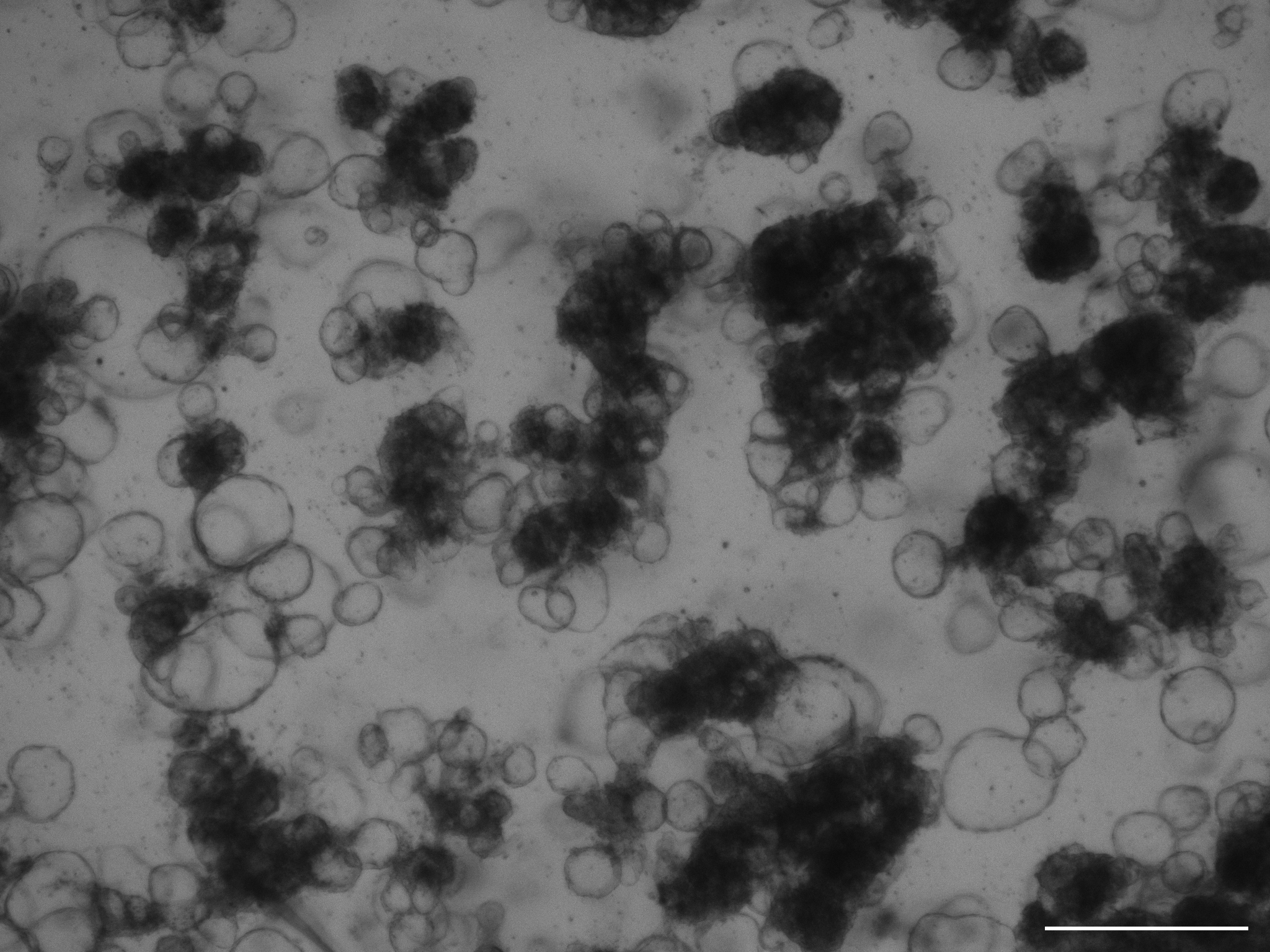

Supplement: Supplementary file 7 — Source data Fig. 4 [file 44321_2025_330_MOESM7_ESM.zip › Figure 4/4M/Brightfield microscopy picture HPDO.tiff]

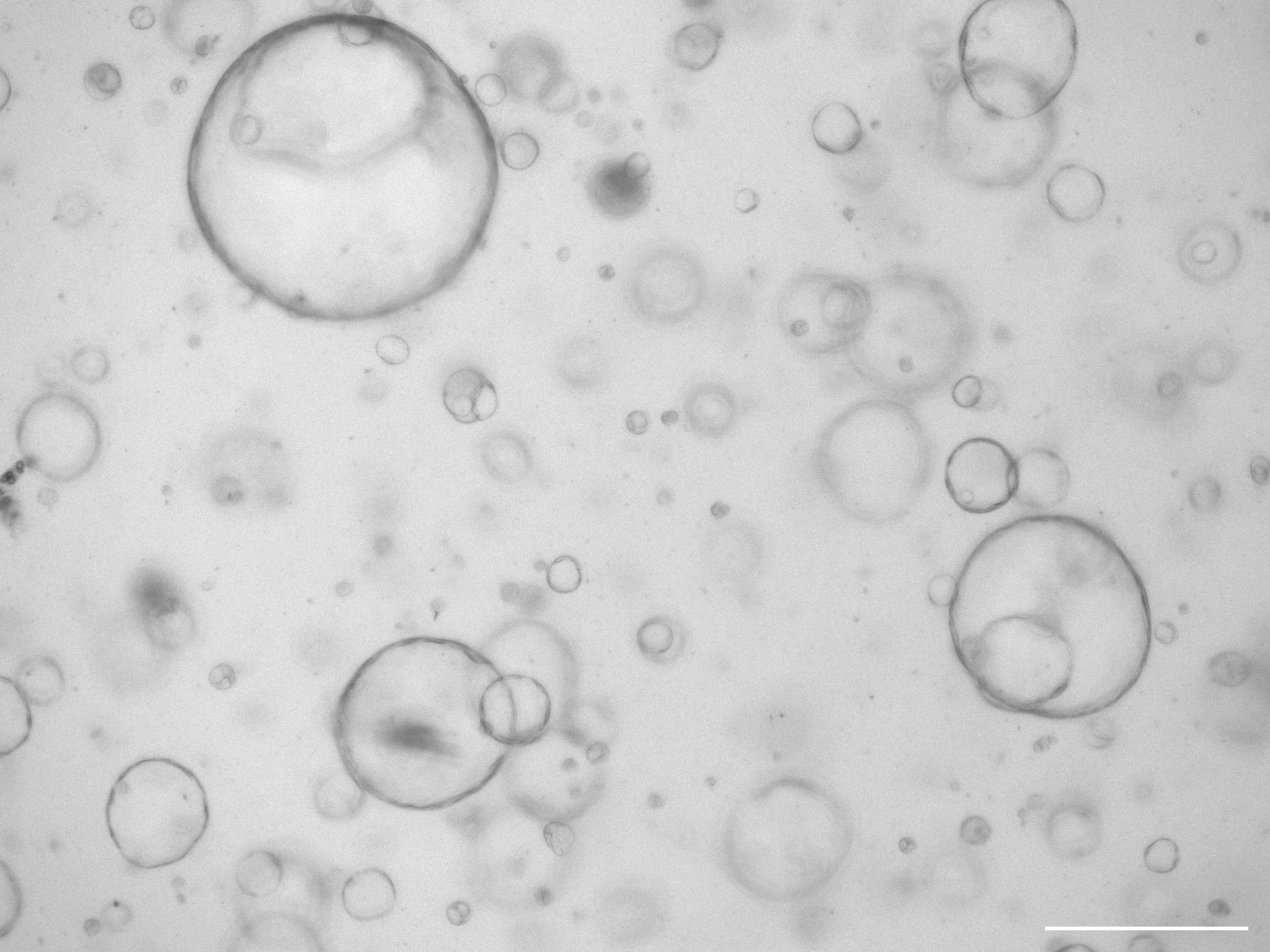

Supplement: Supplementary file 7 — Source data Fig. 4 [file 44321_2025_330_MOESM7_ESM.zip › Figure 4/4N/Brightfield microscopy picture HPDO.tiff]

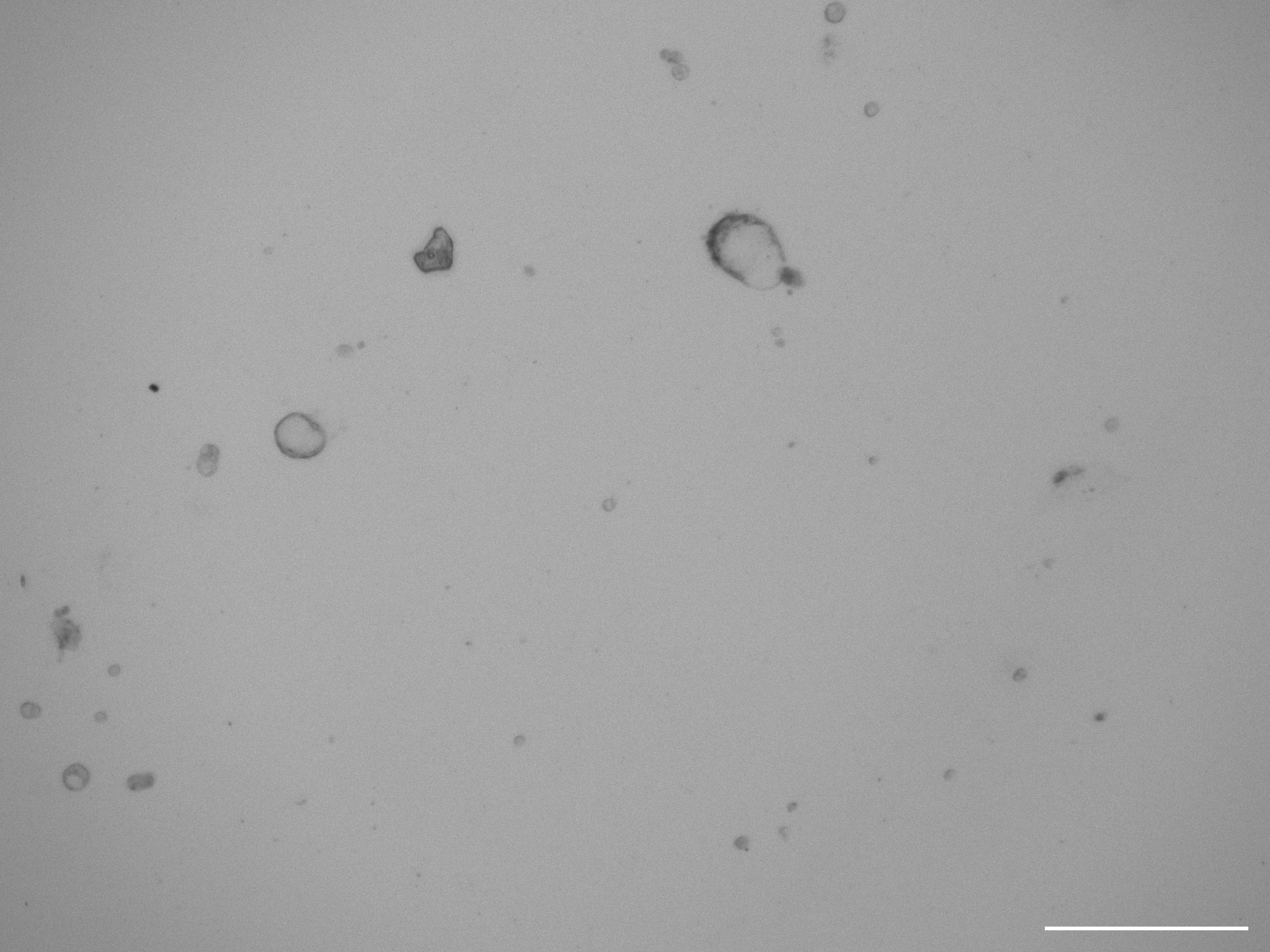

Supplement: Supplementary file 7 — Source data Fig. 4 [file 44321_2025_330_MOESM7_ESM.zip › Figure 4/4O/Brightfield microscopy picture HPDO.tiff]

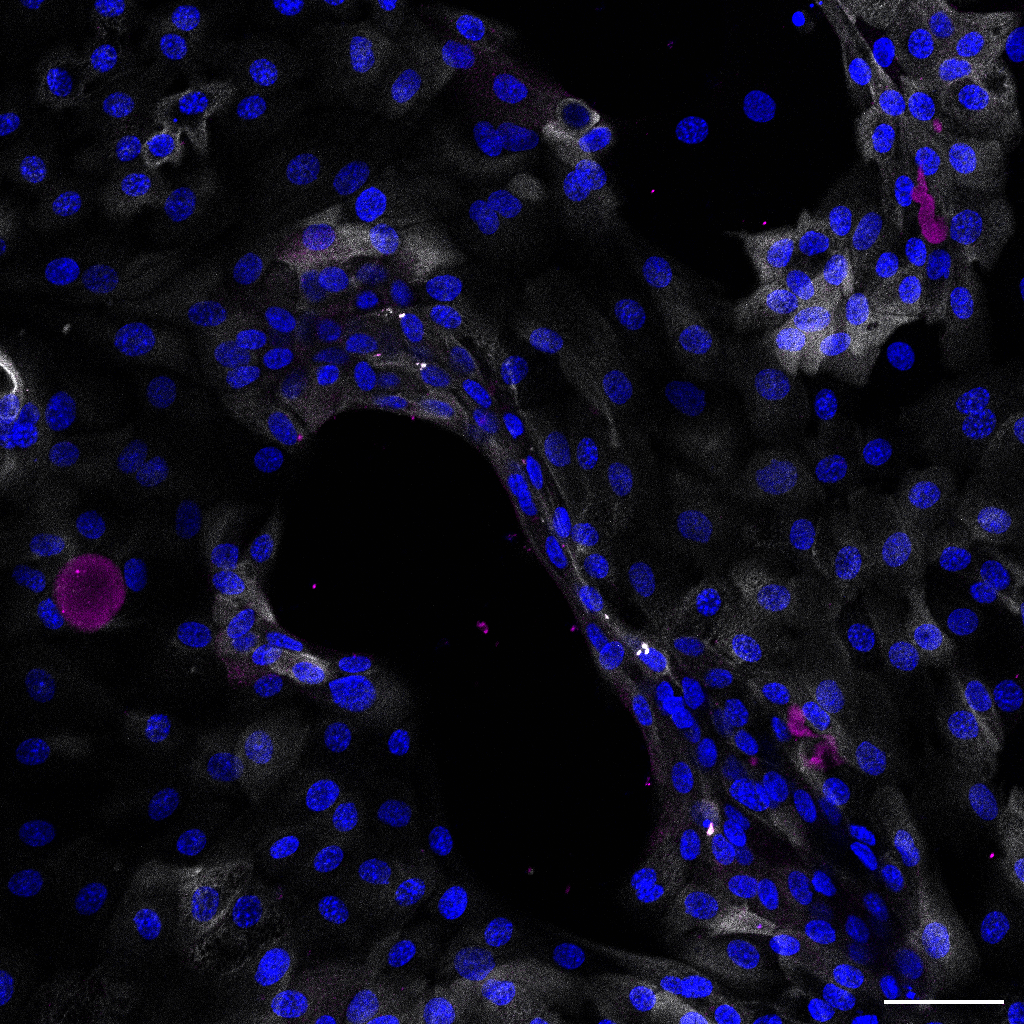

Supplement: Supplementary file 8 — Source data Fig. 5 [file 44321_2025_330_MOESM8_ESM.zip › Figure 5/5H/Confocal microscopy merge image single plane.tif]

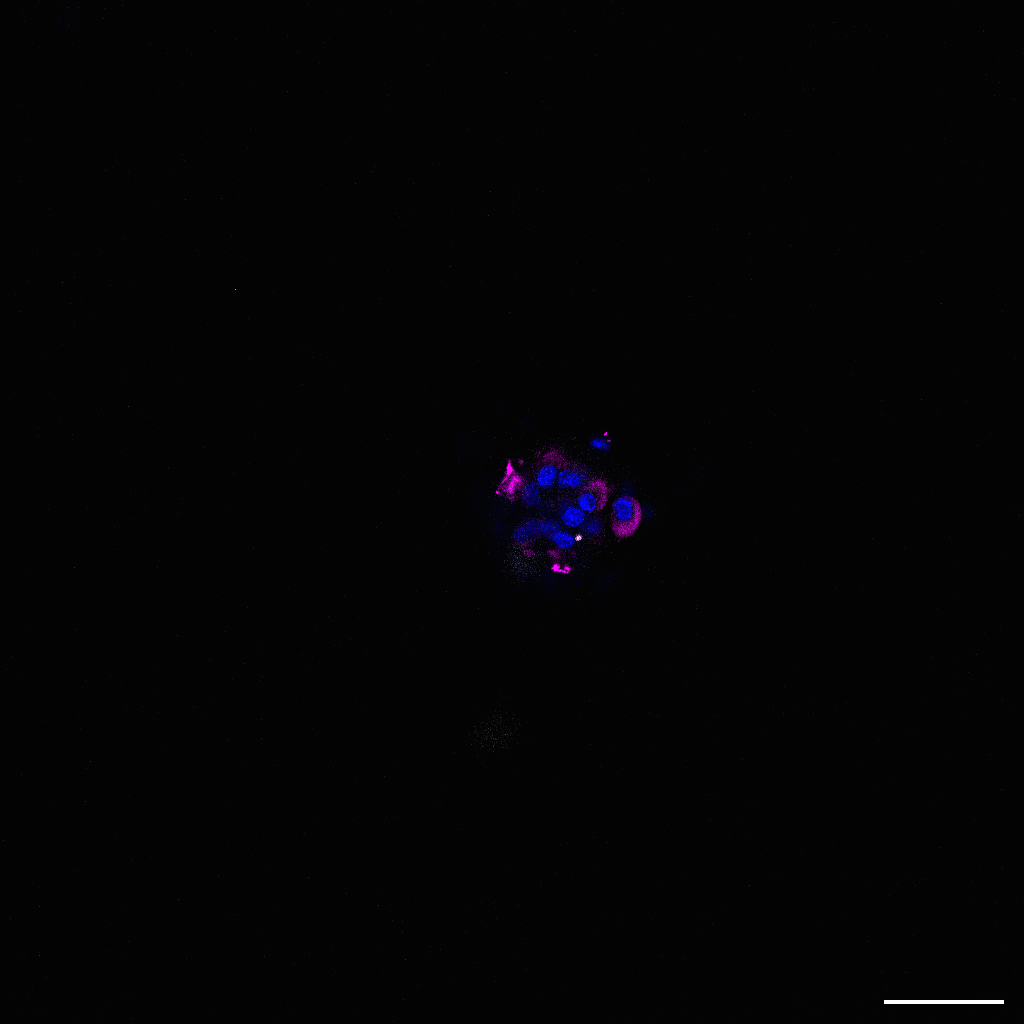

Supplement: Supplementary file 8 — Source data Fig. 5 [file 44321_2025_330_MOESM8_ESM.zip › Figure 5/5I/Confocal microscopy merge image single plane.tif]

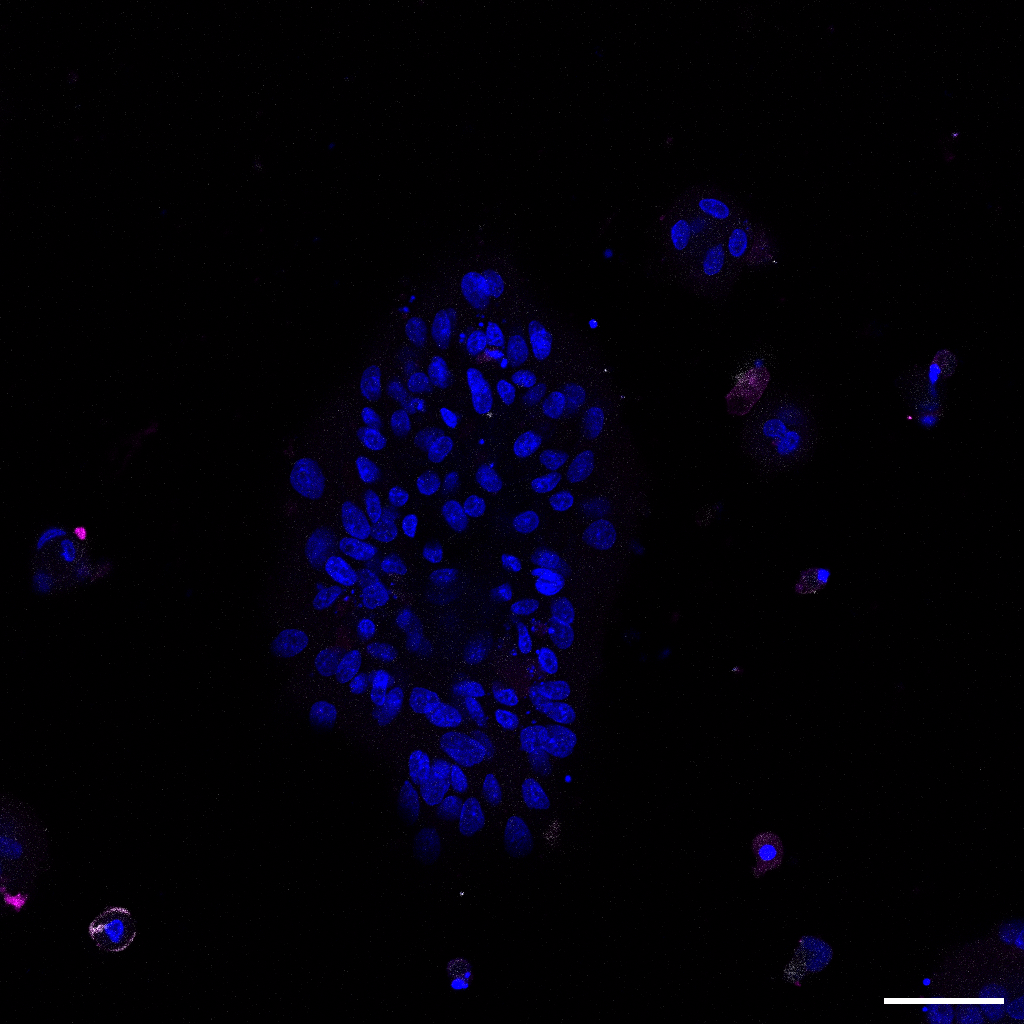

Supplement: Supplementary file 8 — Source data Fig. 5 [file 44321_2025_330_MOESM8_ESM.zip › Figure 5/5J/Confocal microscopy image single plane merge.tif]

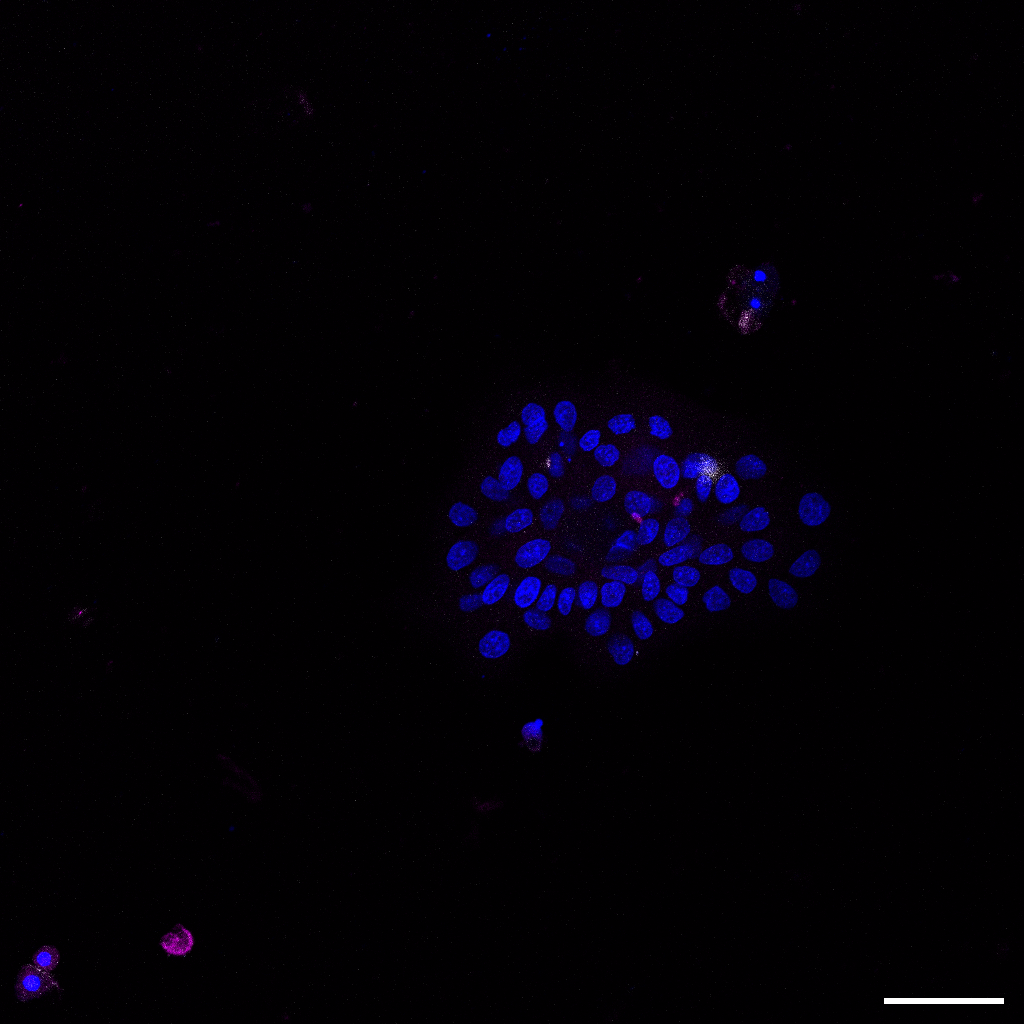

Supplement: Supplementary file 8 — Source data Fig. 5 [file 44321_2025_330_MOESM8_ESM.zip › Figure 5/5K/Confocal microscopy merge image single plane.tif]

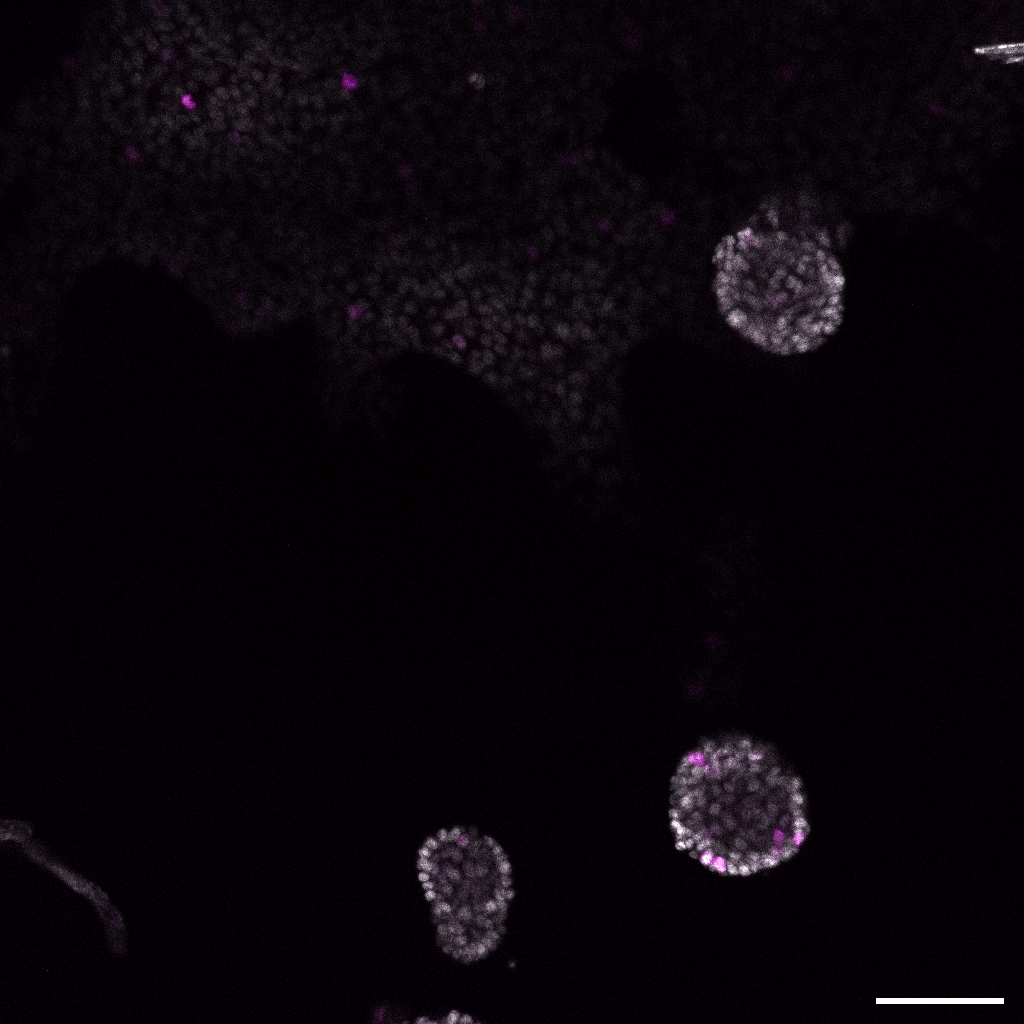

Supplement: Supplementary file 9 — Source data Fig. 6 [file 44321_2025_330_MOESM9_ESM.zip › Figure 6/6D/Confocal microscopy merge image single plane 6d.tif]

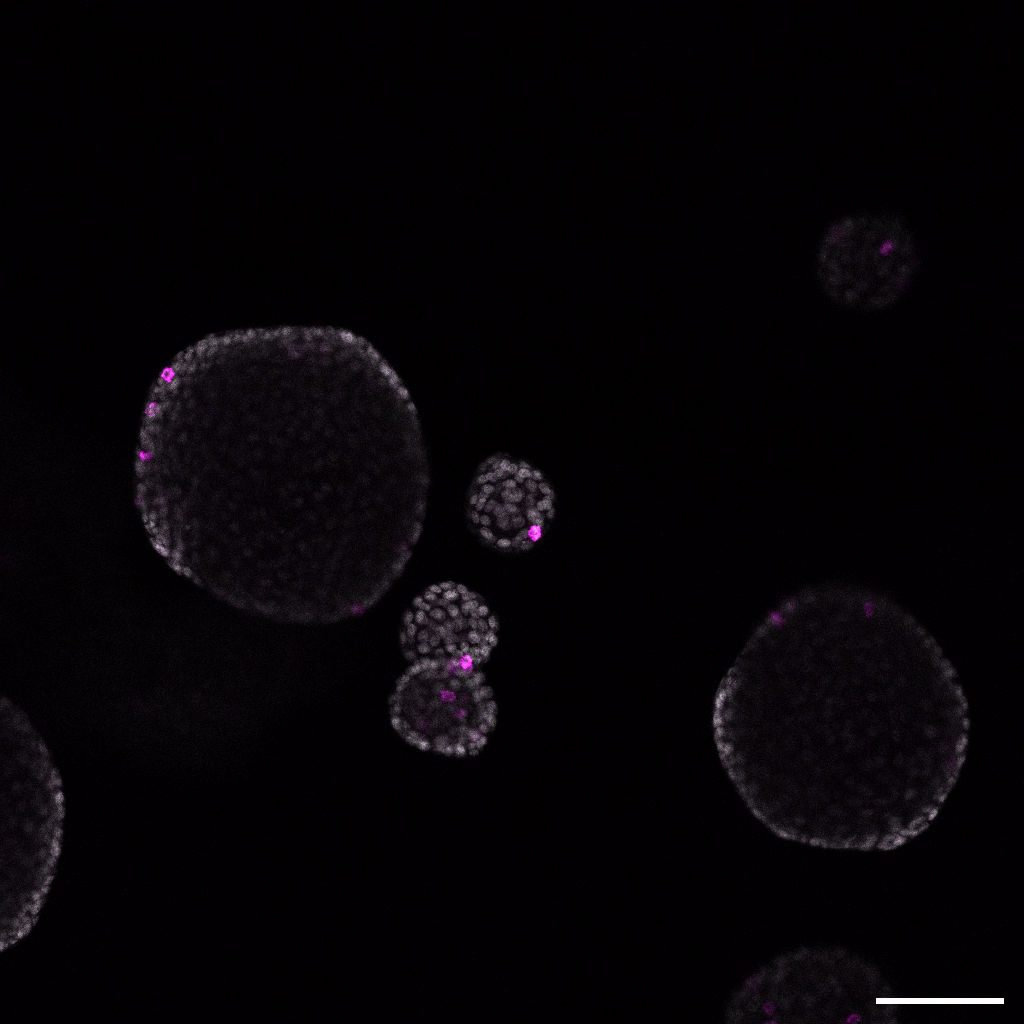

Supplement: Supplementary file 9 — Source data Fig. 6 [file 44321_2025_330_MOESM9_ESM.zip › Figure 6/6E/Confocal microscopy merge image single plane 6E.tif]

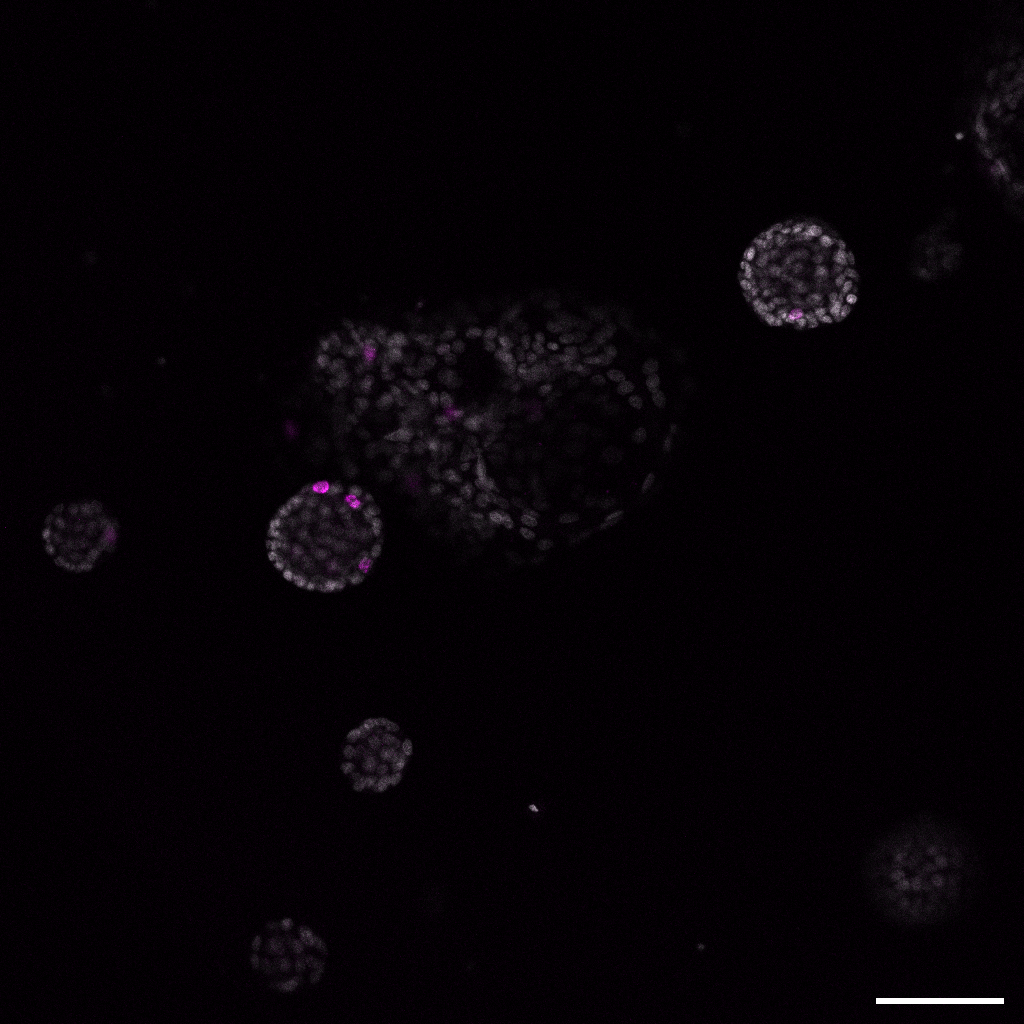

Supplement: Supplementary file 9 — Source data Fig. 6 [file 44321_2025_330_MOESM9_ESM.zip › Figure 6/6F/Confocal microscopy merge image single plane 6F.tif]

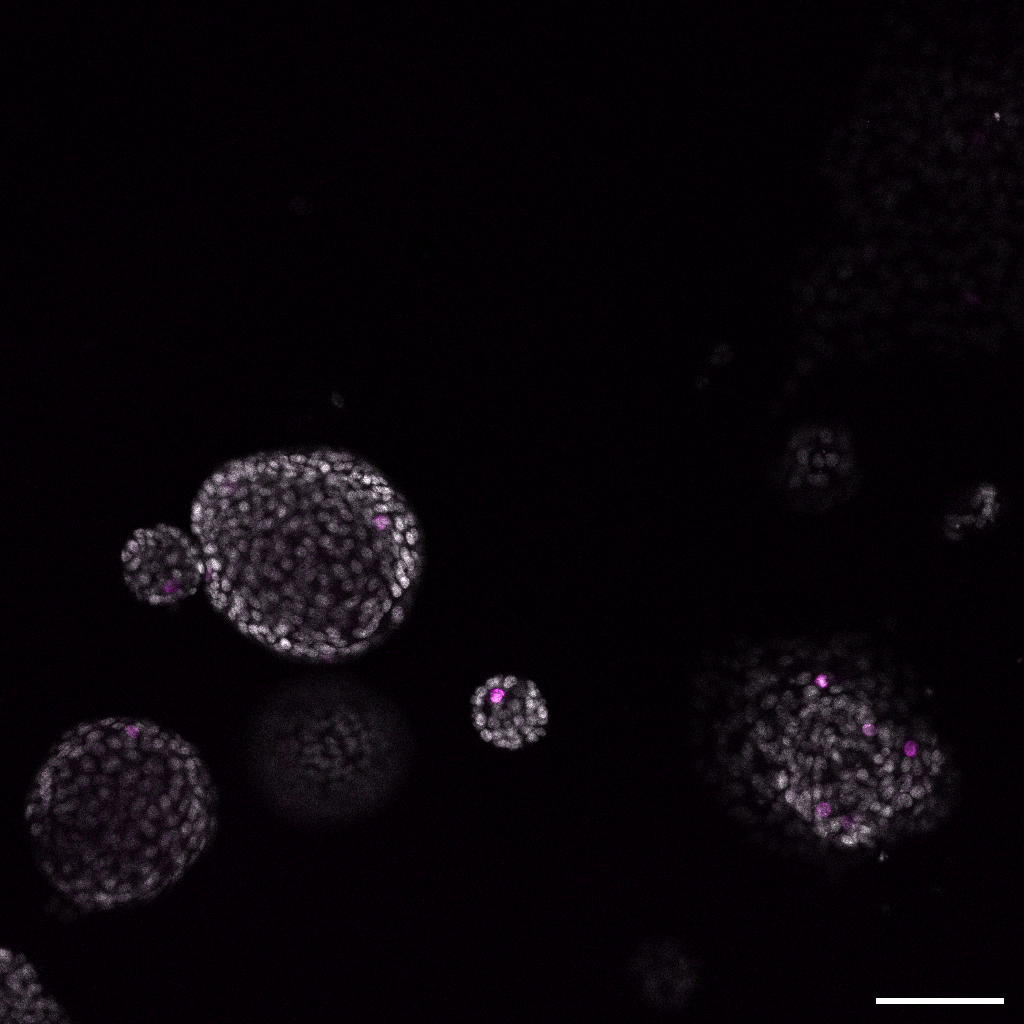

Supplement: Supplementary file 9 — Source data Fig. 6 [file 44321_2025_330_MOESM9_ESM.zip › Figure 6/6G/Confocal microscopy merge image single plane 6G.tif]

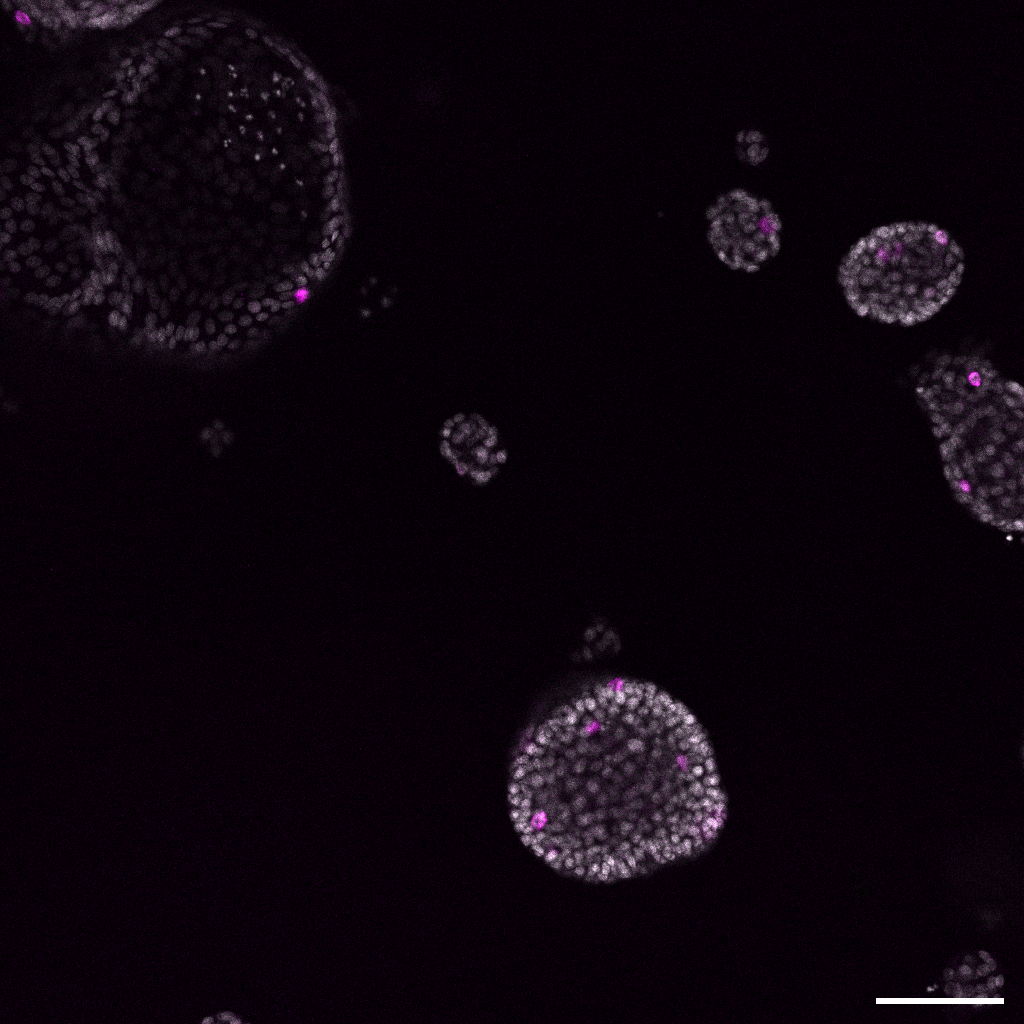

Supplement: Supplementary file 9 — Source data Fig. 6 [file 44321_2025_330_MOESM9_ESM.zip › Figure 6/6H/Confocal microscopy merge image single plane 6H.tif]

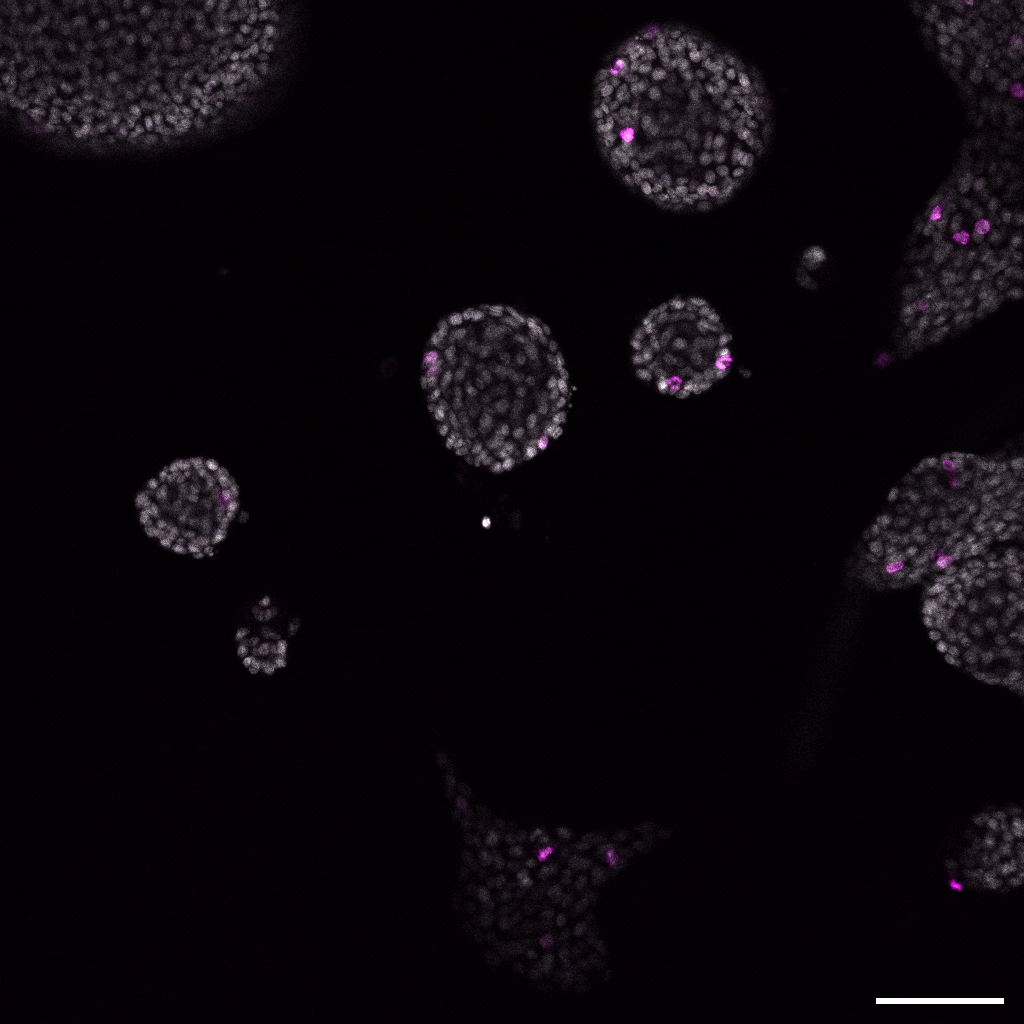

Supplement: Supplementary file 9 — Source data Fig. 6 [file 44321_2025_330_MOESM9_ESM.zip › Figure 6/6I/Confocal microscopy merge image single plane 6I.tif]

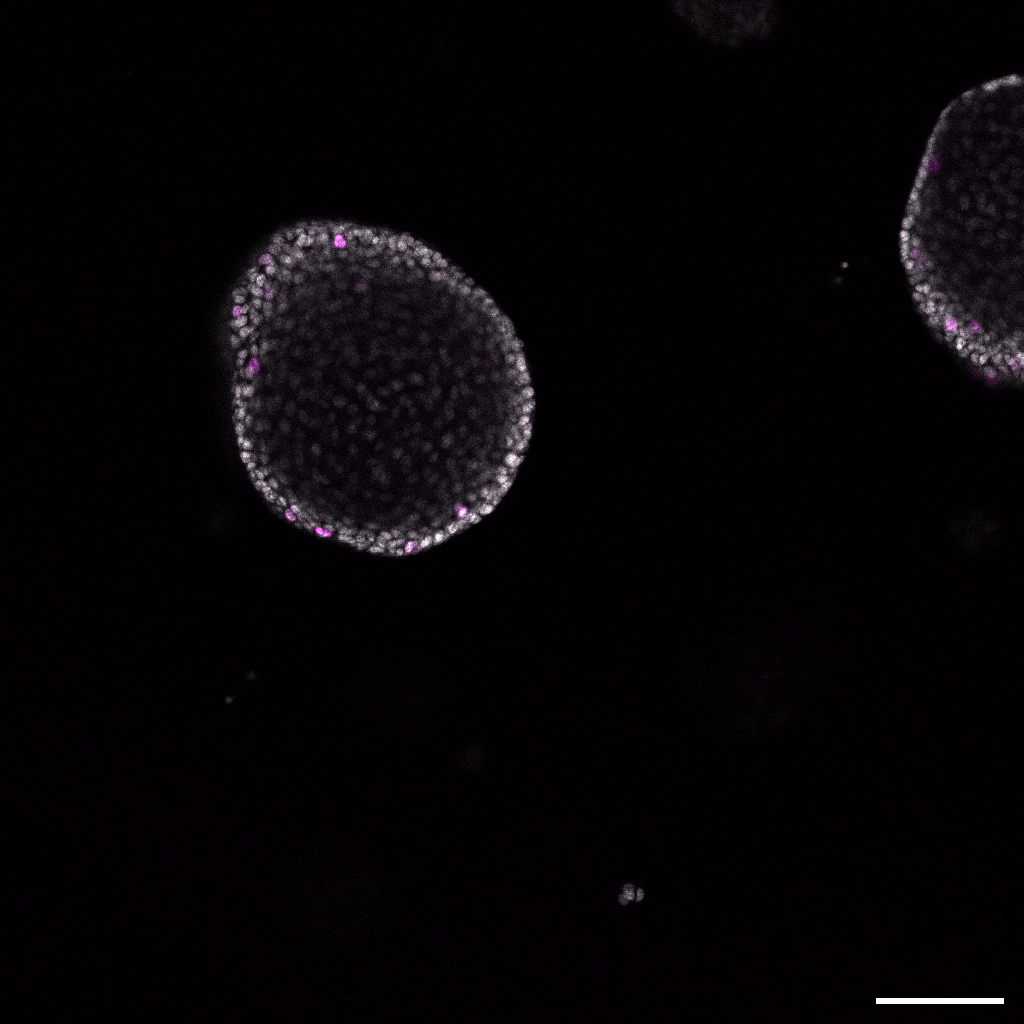

Supplement: Supplementary file 9 — Source data Fig. 6 [file 44321_2025_330_MOESM9_ESM.zip › Figure 6/6J/Confocal microscopy merge image single plane 6J.tif]

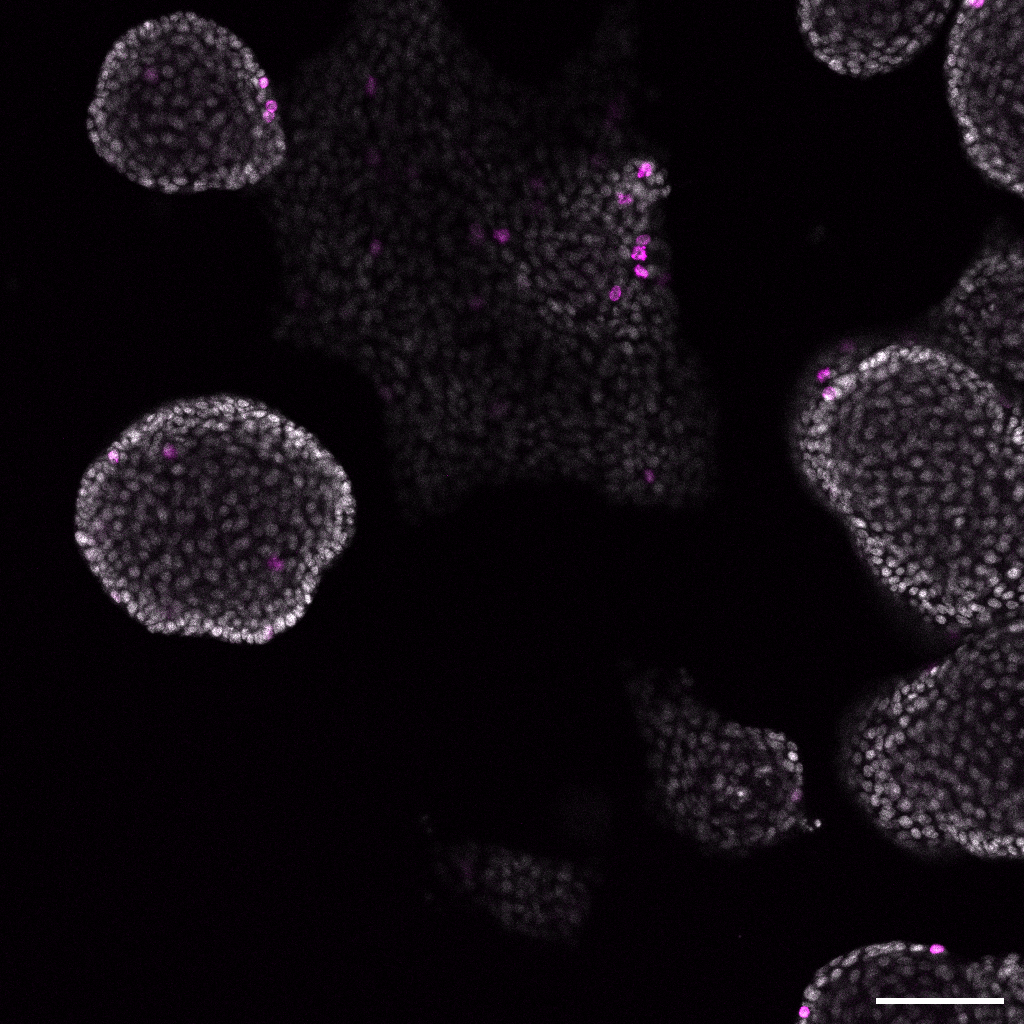

Supplement: Supplementary file 9 — Source data Fig. 6 [file 44321_2025_330_MOESM9_ESM.zip › Figure 6/6K/Confocal microscopy merge image single plane 6K.tif]

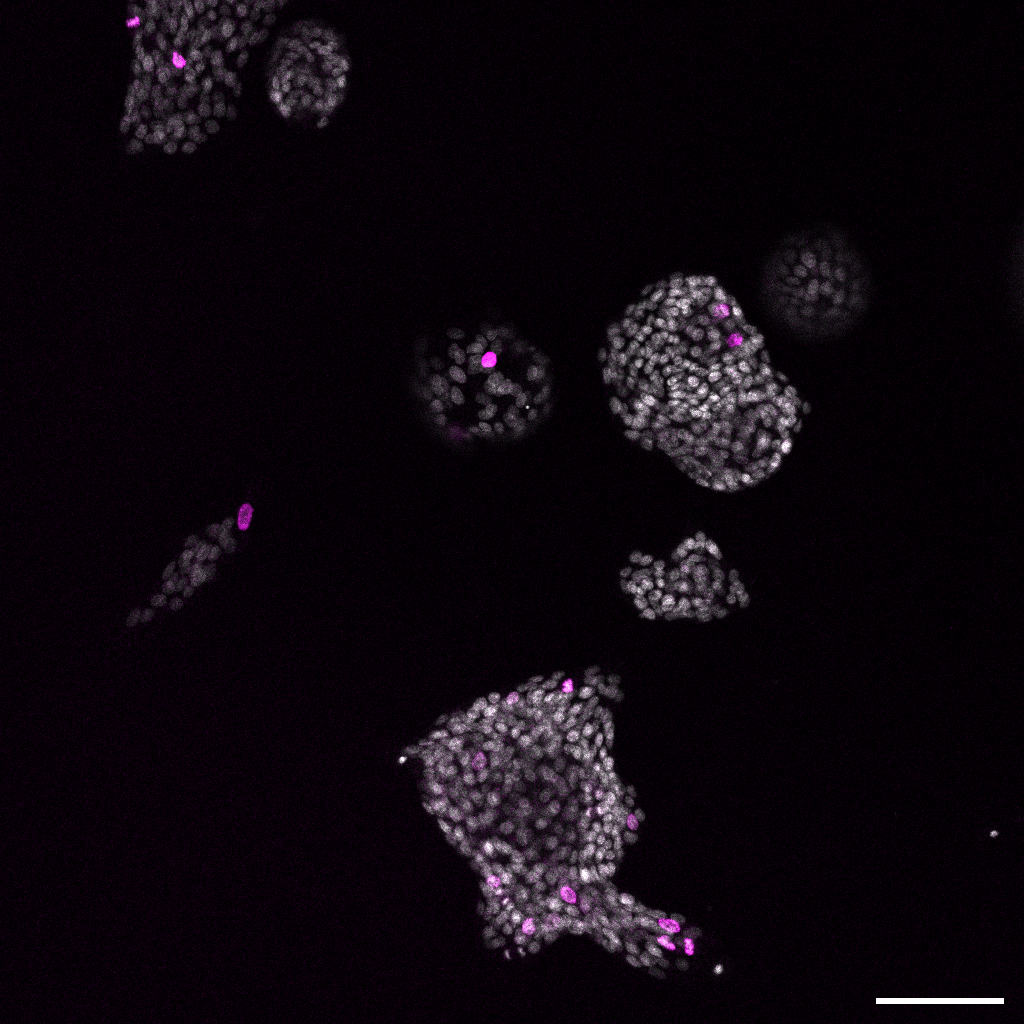

Supplement: Supplementary file 9 — Source data Fig. 6 [file 44321_2025_330_MOESM9_ESM.zip › Figure 6/6L/Confocal microscopy merge image single plane 6L.tif]

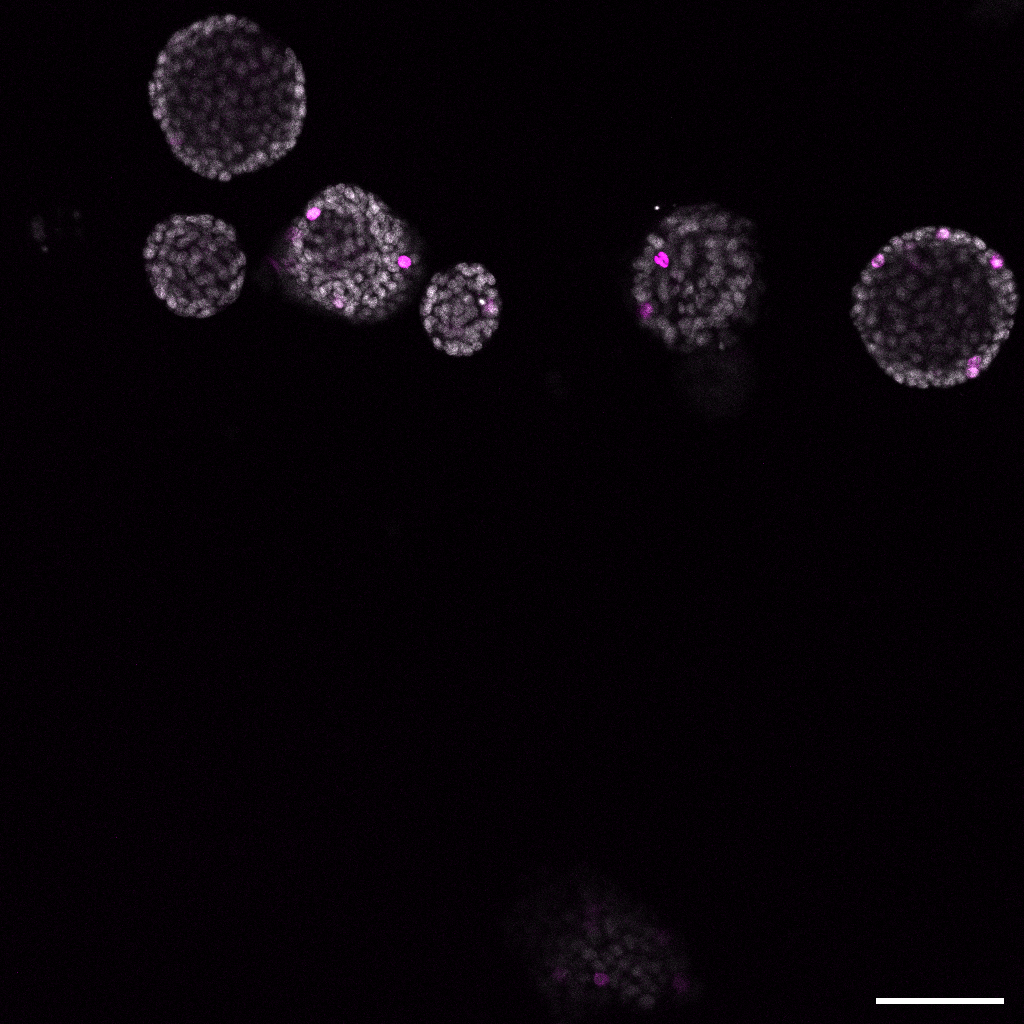

Supplement: Supplementary file 9 — Source data Fig. 6 [file 44321_2025_330_MOESM9_ESM.zip › Figure 6/6M/Confocal microscopy merge image single plane 6M.tif]
