# Supplementary material for: Benchmarking porcine pancreatic ductal organoids for drug screening applications
Source: EMBO Mol Med. 2025 Nov 4;17(12):3657–88. doi: 10.1038/s44321-025-00330-3 (PMC12686538; doi:10.1038/s44321-025-00330-3)
Supplement: Supplementary file 10 — Expanded View Figures [file 44321_2025_330_MOESM10_ESM.pdf]

Expanded View Figures

Figure EV1. Pancreatic marker expression in PPDO from different developmental stages.

(**A–C**) Single-plane confocal images of PPDO derived from one Em pig pancreas immunostained against Pan-Cytokeratin (PAN-CK)-BMPR1A-CDH1 (**A**), SOX9-Phalloidin-NKX6-1 (**B**), CFTR-GP2-PDX1 (**C**) and counterstained with DAPI. Insets show the individual channels of the merge image. PPDO were stained at passage 2. Scale bar: 50 μm. (**D–F**) Single-plane confocal images of PPDO derived from an LPN pig pancreas immunostained against KRT5-BMPR1A-CDH1 (**D**), SOX9-GCG/NKX6-1-Phalloidin (**E**), CFTR-GP2-PDX1 (**F**) and counterstained with DAPI. Insets show the individual channels of the merge image. PPDO were stained at passage 4. Scale bar: 50 μm. (**G–I**) Single-plane confocal images of PPDO derived from an Ad pig pancreas immunostained against CFTR-GP2-PDX1 (**G**), SOX9-Phalloidin-CDH1 (**H**), AMY3A-KRT7-NEUROG3 (**I**), and counterstained with DAPI. PPDO were stained at passage 3. Insets show the individual channels of the merge image. Scale bar: 50 μm. (**J–L**) Single-plane confocal images of HPDO immunostained against KRT7-AGR2-CDH1 (**J**), CFTR-BMPR1A-NKX6.1 (**K**), SOX9-Phalloidin-PDX1 (**L**), and counterstained with DAPI. Insets show the individual channels of the merge image. HPDO were stained at passage 3. Scale bar: 50 μm. (**M**, **N**) Single-plane confocal images of porcine pancreas cryosections (Ad) immunostained against NKX6.1-SOX9-CDH1 (**M**) and phalloidin-555-PDX1 (**N**), and counterstained with DAPI. Scale bar: 50 μm. (**O**) Quantification of the organoid area of late passage (>5) PPDO following 4 h of live imaging and treatment with forskolin. $n = 3$ independent experiments with 3 PPDO lines (2 EPN and 1 Ad). Data are shown as mean ± SD. One-way ANOVA followed by Dunnett's multiple comparisons test was used to assess significance. Not-significant (ns) $P = 0.9010$.

 © The Author(s)

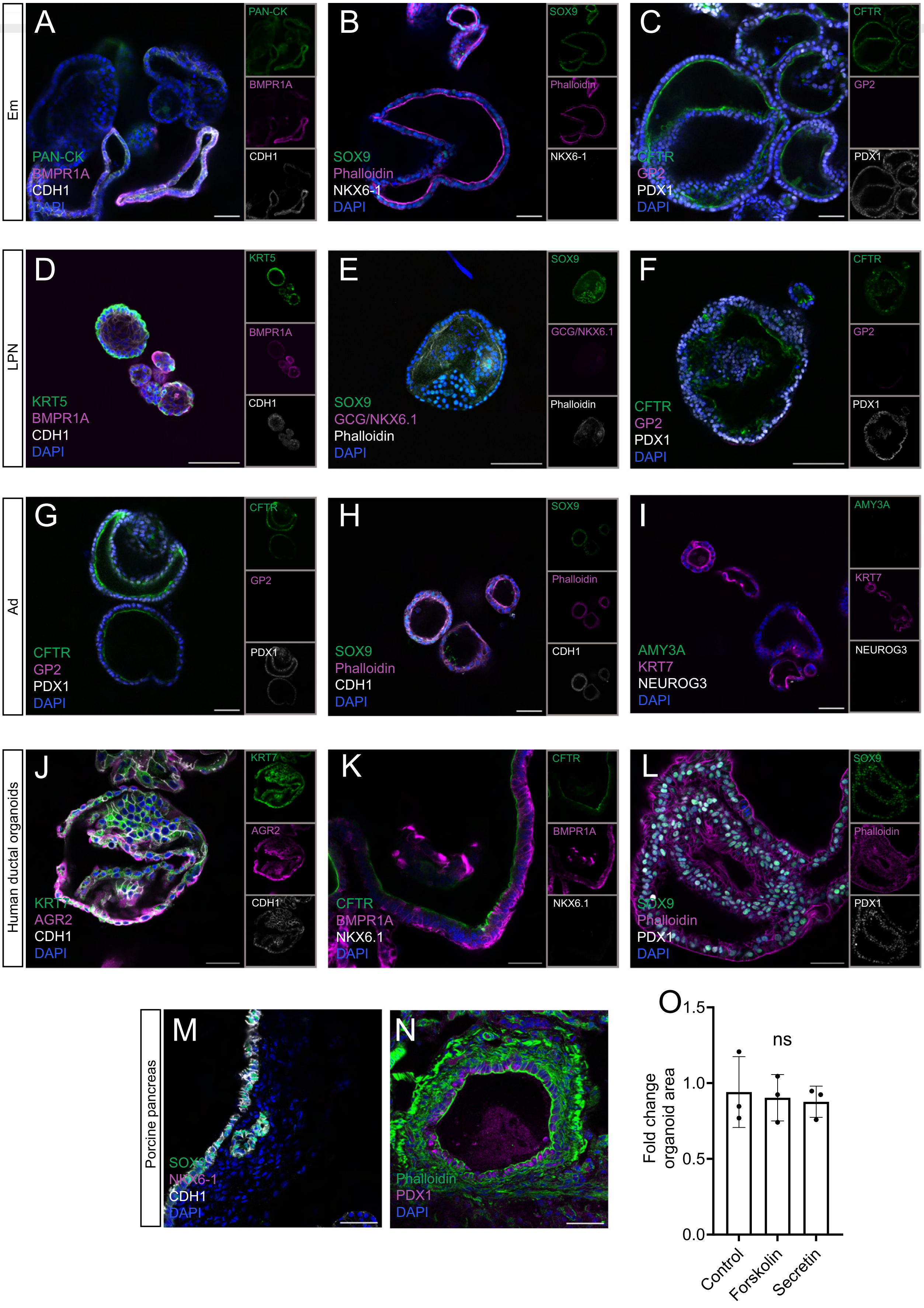
Em
A
PAN-CK
BMPR1A
CDH1
DAPI
B
SOX9
Phalloidin
NKX6-1
DAPI
C
CFTR
GP2
PDX1
DAPI
LPN
D
KRT5
BMPR1A
CDH1
DAPI
E
SOX9
GCG/NKX6.1
Phalloidin
DAPI
F
CFTR
GP2
PDX1
DAPI
Ad
G
CFTR
GP2
PDX1
DAPI
H
SOX9
Phalloidin
CDH1
DAPI
I
AMY3A
KRT7
NEUROG3
DAPI
Human ductal organoids
J
KRT7
AGR2
CDH1
DAPI
K
CFTR
BMPR1A
NKX6.1
DAPI
L
SOX9
Phalloidin
PDX1
DAPI
Porcine pancreas
M
SOX9
NKX6-1
CDH1
DAPI
N
Phalloidin
PDX1
DAPI
O
Fold change organoid area
0.0
0.5
1.0
1.5
ns
Control
Forskolin
Secretin

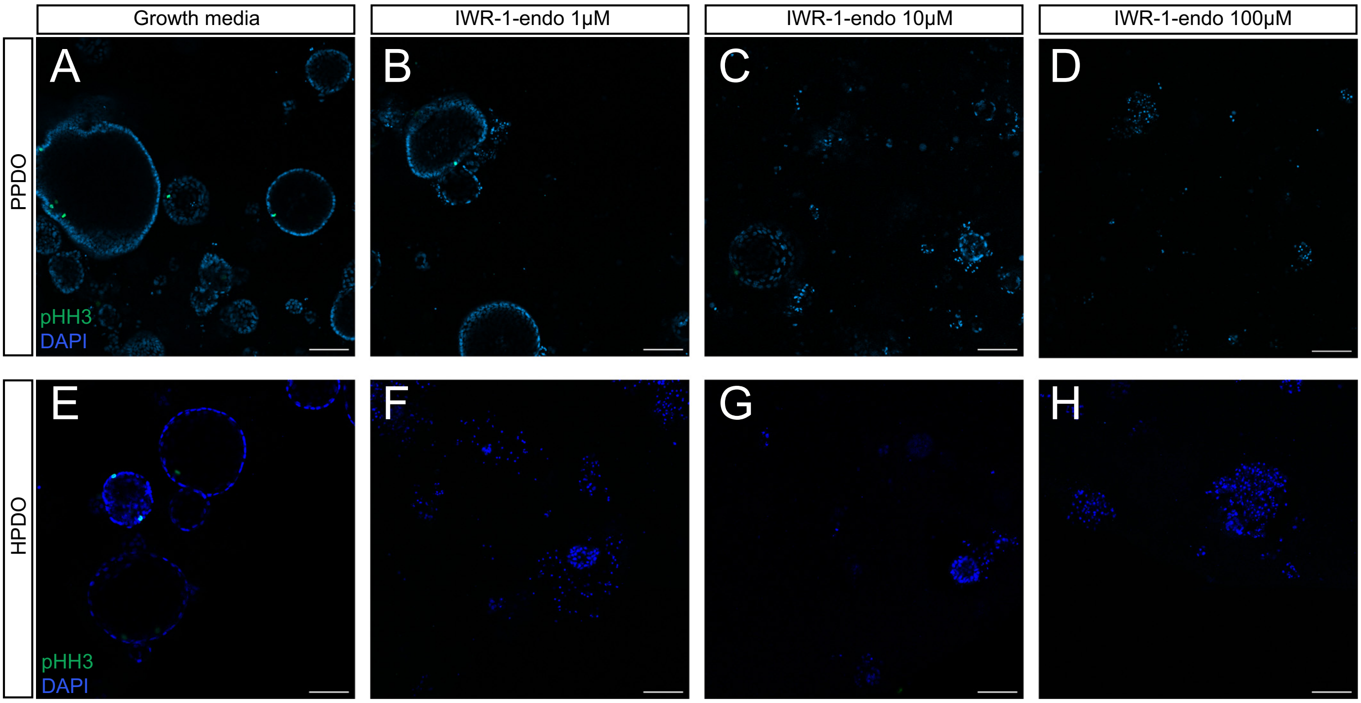

Figure EV2. PPDO and HPDO proliferation arrest upon WNT inhibition.

(**A–D**) Single-plane confocal images of PPDO in growth media (**A**) or in media lacking WNT3a,R-Spondin-1 treated with 1 μM (**B**), 10 μM (**C**) or 100 μM (**D**) IWR-1-endo WNT signaling inhibitor. PPDO were immunostained for pHH3 and counterstained with DAPI. Experiment was repeated with $n = 4$ biological replicates (1 Em, 1 EPN, 2 Ad). Scale bar: 100 μm. (**E–H**) Single-plane confocal images of HPDO in growth media (**E**) or in media lacking WNT3a,R-Spondin-1 treated with 1 μM (**F**), 10 μM (**G**) or 100 μM (**H**) IWR-1-endo WNT signaling inhibitor. HPDO were immunostained for pHH3 and counterstained with DAPI. Experiment was repeated with $n = 3$ biological replicates. Scale bar: 100 μm.

 © The Author(s)

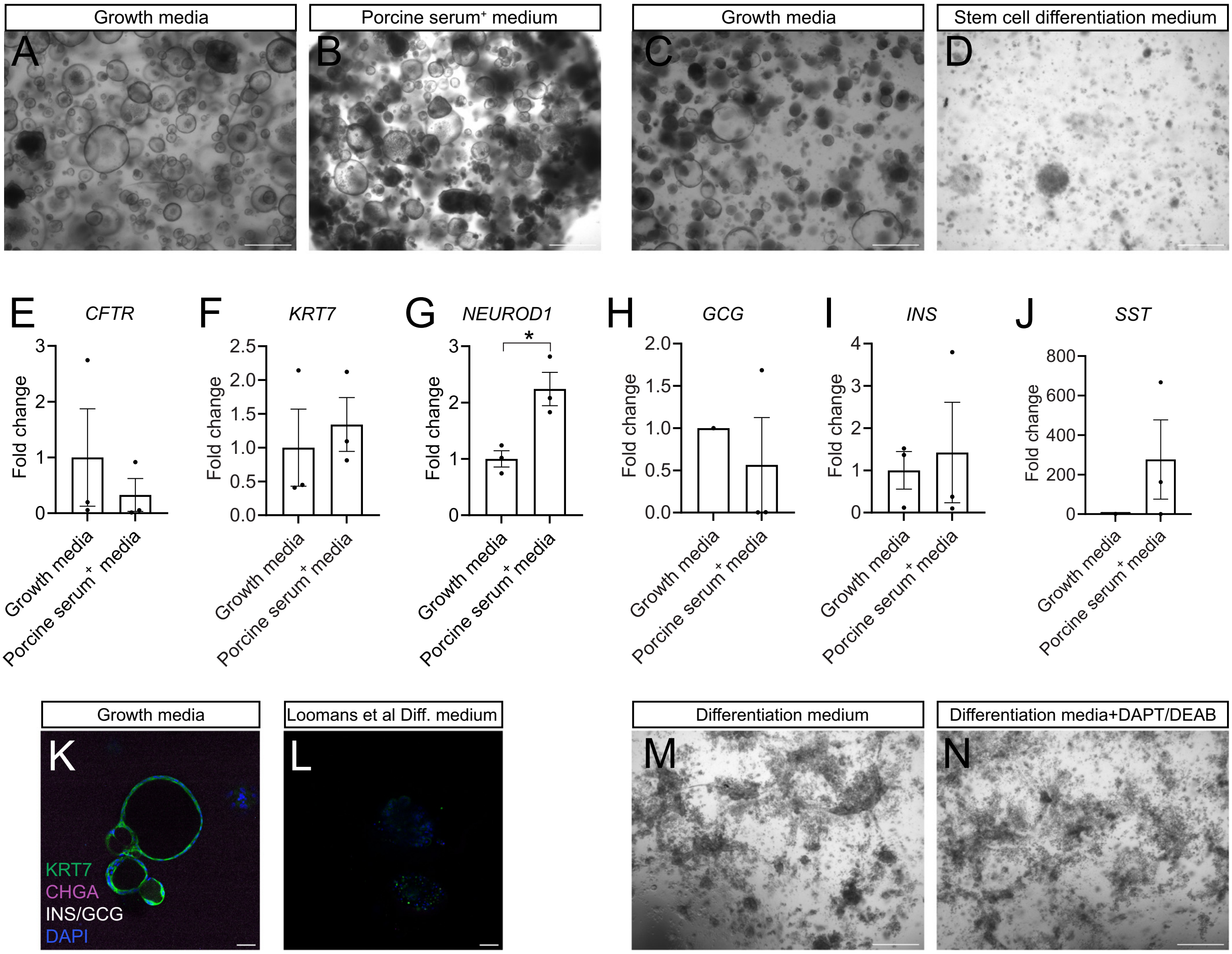

Figure EV3. Endocrine differentiation protocol testing.

(**A**, **B**) Brightfield microscopy images of PPDO in complete (**A**) or porcine serum supplemented (**B**). Scale bar 500 µm. (**C**, **D**) Brightfield microscopy images of PPDO at the end of differentiation in complete (**C**) or at the end of the differentiation using S5 + S6 combination (**D**) media. Scale bar 500 µm. (**E–J**) Bar plots showing the fold change of gene expression analysis at the end of the differentiation after treatment with porcine serum. Gene expression was measured for *CFTR* (**E**), *KRT7* (**F**), *NEUROD1* (**G**), *GCG* (**H**), *INS* (**I**), and *SST* (**J**). $n = 3$ independent PPDO lines (1 Em and 2 EPN). Absence of samples from the plots indicate non-detectable amplification following the RT-PCR. Data are shown as mean ± SEM. Unpaired Student's t-test was used to assess significance with $^{*}P = 0.0196$ for *NEUROD1*. (**K**, **L**) Single-plane confocal images of PPDO in growth media (**K**) or in differentiation media from Loomans et al (**L**) (see Methods). PPDO were immunostained against INS-KRT7-CHGA and counterstained with DAPI. Experiment was repeated with $n = 3$ biological replicates (1 Em-1 EPN-1 Ad). Scale bar: 50 µm. (**M**, **N**) Brightfield microscopy images of HPDO in differentiation media (**M**) or differentiation media supplemented with DAPT/DEAB small molecules (**N**). Experiment was repeated with $n = 3$ biological replicates. Scale bar 500 µm.

© The Author(s)

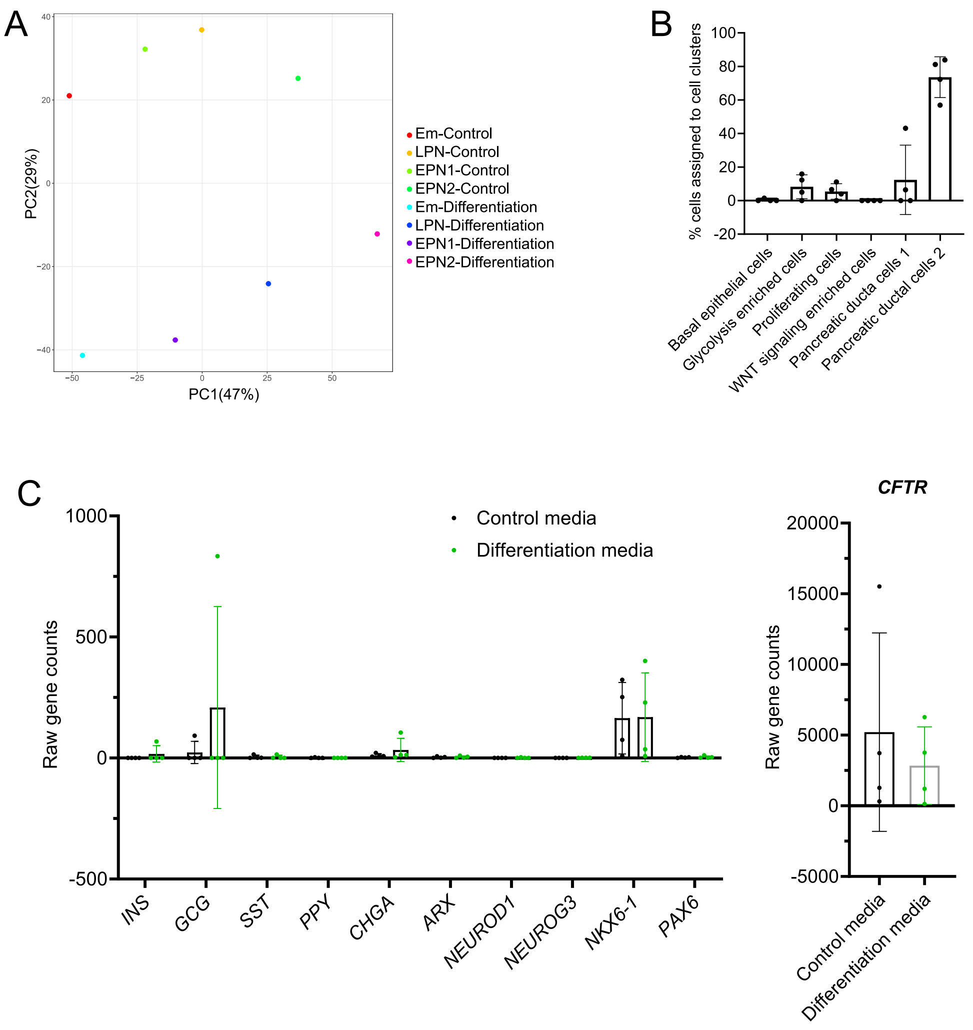

Figure EV4. Quality assessment of the RNA-Seq differentiation analysis.

(**A**) PCA plot of the bulk RNA-Seq results from the differentiation test showing the first two components. (**B**) Bar plot showing the percentage for each cell type found of the PPDO scRNA-Seq dataset in each of the 4 biological replicates of the bulk RNA-Seq dataset. Data show the mean ± SD. (**C**) Bar plot showing the raw counts (gene length scaled) of the RNA-Seq dataset for control and differentiation media. Each dot represents each of the biological replicate PPDO sequenced from 1 Em, 2 EPN and 1 LPN samples ($n = 4$). Data show the mean ± SD.

 © The Author(s)

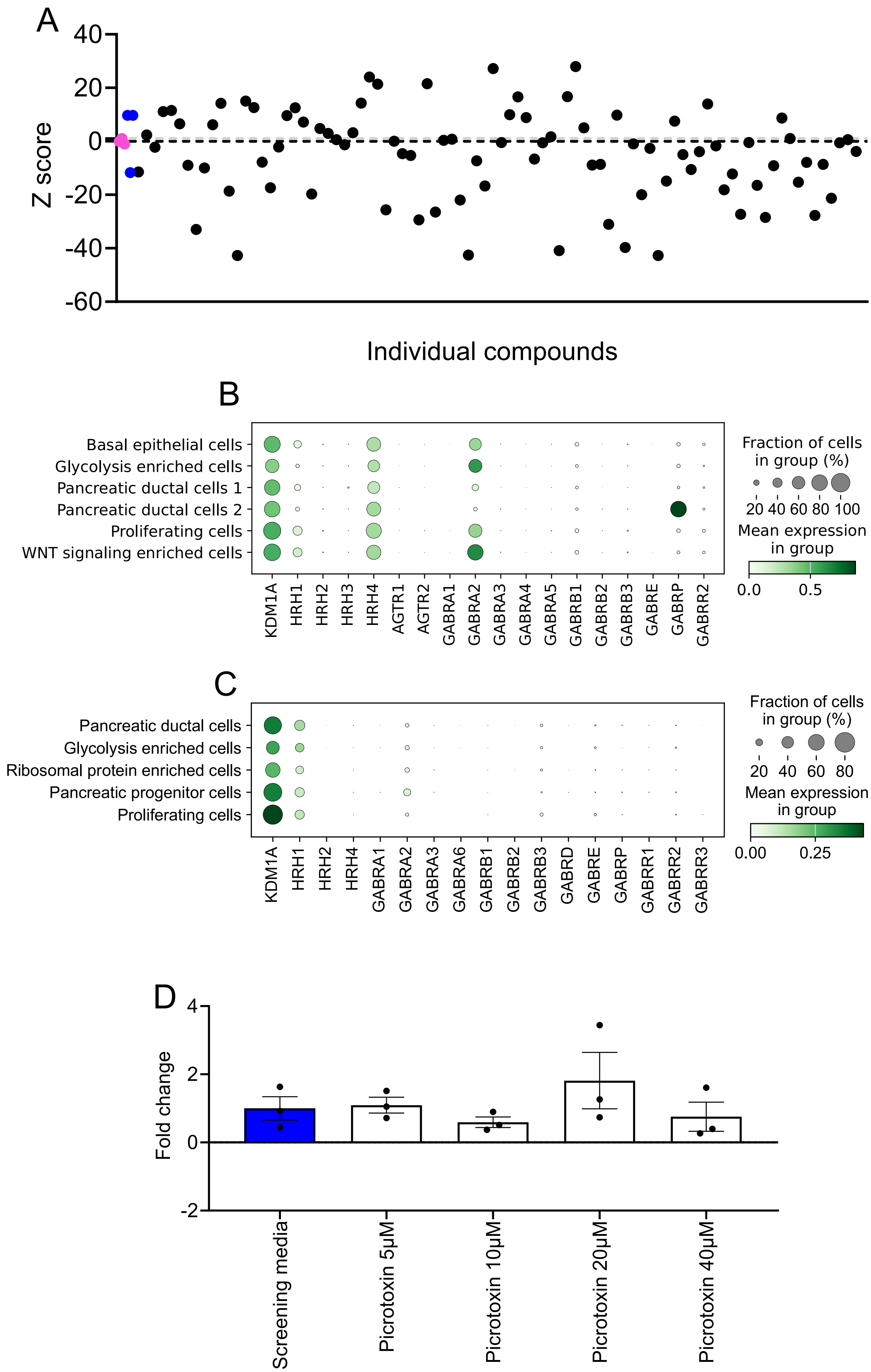
A
Z score
40
20
0
-20
-40
-60
Individual compounds
B
Basal epithelial cells
Glycolysis enriched cells
Pancreatic ductal cells 1
Pancreatic ductal cells 2
Proliferating cells
WNT signaling enriched cells
KDM1A HRH1 HRH2 HRH3 HRH4 AGTR1 AGTR2 GABRA1 GABRA2 GABRA3 GABRA4 GABRA5 GABRB1 GABRB2 GABRB3 GABRE GABRP GABRR2
Fraction of cells in group (%)
20 40 60 80 100
Mean expression in group
0.0 0.5
C
Pancreatic ductal cells
Glycolysis enriched cells
Ribosomal protein enriched cells
Pancreatic progenitor cells
Proliferating cells
KDM1A HRH1 HRH2 HRH4 GABRA1 GABRA2 GABRA3 GABRA6 GABRB1 GABRB2 GABRB3 GABRD GABRE GABRP GABRR1 GABRR2 GABRR3
Fraction of cells in group (%)
20 40 60 80
Mean expression in group
0.00 0.25
D
Fold change
4
2
0
-2
Screening media
Picrotoxin 5μM
Picrotoxin 10μM
Picrotoxin 20μM
Picrotoxin 40μM

© The Author(s)

Figure EV5. Validating primary chemical screen data.

(**A**) Scatter plots reporting the z-scores of the ATP-luciferase assay of the primary screen results compared to the complete medium control wells for one PPDO line (Em as in main figure). Each dot represents a single well of the 96-well plate treated with a single chemical. Magenta dots show the individual wells of PPDOs treated in the complete medium with DMSO (control) and blue dots show the individual wells of PPDOs treated in screening medium (complete medium -WRN) with DMSO and the line for these two wells corresponding to the median of the 3 wells/condition. Gray dotted line demarcates z-score of 1, black dotted line a z-score of 0. (**B**, **C**) Dot plots showing gene expression of the genes in the pathways targeted by the primary hits of the chemical screen in the PPDO (**B**) and HPDO (**C**) scRNA-Seq dataset. (**D**) Bar plot reporting the fold change of the proliferation percentage of the HPDO dose-response experiment with picrotoxin treatment. $n = 3$ individual HPDO lines. Error bars show the ±SEM. Not significant as assessed by a Kruskal–Wallis statistical test.

 © The Author(s)
